# Barcoding and Border Biosecurity: Identifying Cyprinid Fishes in the Aquarium Trade

**DOI:** 10.1371/journal.pone.0028381

**Published:** 2012-01-20

**Authors:** Rupert A. Collins, Karen F. Armstrong, Rudolf Meier, Youguang Yi, Samuel D. J. Brown, Robert H. Cruickshank, Suzanne Keeling, Colin Johnston

**Affiliations:** 1 Bio-Protection Research Centre, Lincoln University, Canterbury, New Zealand; 2 Department of Biological Sciences and University Scholars Programme, National University of Singapore, Singapore, Singapore; 3 Department of Ecology, Faculty of Agriculture and Life Sciences, Lincoln University, Canterbury, New Zealand; 4 Animal Health Laboratory, Investigation and Diagnostic Centre, Ministry of Agriculture and Forestry, Upper Hutt, New Zealand; Biodiversity Insitute of Ontario - University of Guelph, Canada

## Abstract

**Background:**

Poorly regulated international trade in ornamental fishes poses risks to both biodiversity and economic activity via invasive alien species and exotic pathogens. Border security officials need robust tools to confirm identifications, often requiring hard-to-obtain taxonomic literature and expertise. DNA barcoding offers a potentially attractive tool for quarantine inspection, but has yet to be scrutinised for aquarium fishes. Here, we present a barcoding approach for ornamental cyprinid fishes by: (1) expanding current barcode reference libraries; (2) assessing barcode congruence with morphological identifications under numerous scenarios (e.g. inclusion of GenBank data, presence of singleton species, choice of analytical method); and (3) providing supplementary information to identify difficult species.

**Methodology/Principal Findings:**

We sampled 172 ornamental cyprinid fish species from the international trade, and provide data for 91 species currently unrepresented in reference libraries (GenBank/Bold). DNA barcodes were found to be highly congruent with our morphological assignments, achieving success rates of 90–99%, depending on the method used (neighbour-joining monophyly, bootstrap, nearest neighbour, GMYC, percent threshold). Inclusion of data from GenBank (additional 157 spp.) resulted in a more comprehensive library, but at a cost to success rate due to the increased number of singleton species. In addition to DNA barcodes, our study also provides supporting data in the form of specimen images, morphological characters, taxonomic bibliography, preserved vouchers, and nuclear rhodopsin sequences. Using this nuclear rhodopsin data we also uncovered evidence of interspecific hybridisation, and highlighted unrecognised diversity within popular aquarium species, including the endangered Indian barb *Puntius denisonii*.

**Conclusions/Significance:**

We demonstrate that DNA barcoding provides a highly effective biosecurity tool for rapidly identifying ornamental fishes. In cases where DNA barcodes are unable to offer an identification, we improve on previous studies by consolidating supplementary information from multiple data sources, and empower biosecurity agencies to confidently identify high-risk fishes in the aquarium trade.

## Introduction

Globalisation in the form of international trade breaches biogeographical as well as administrative boundaries, enabling organisms to colonise regions beyond their contemporaneous natural ranges [1]. The impacts of invasive alien species are well documented as a leading cause of global biodiversity decline and economic loss [2], [3], and particularly as a driving force in the biotic homogenisation and degradation of freshwater ecosystems [4]–[6]. Biosecurity challenges exist in effectively monitoring and managing the complex pathways involved [1], [7], [8], with a key issue for risk assessment being the identification of traded biological materials to species [9]–[11]. Effective cataloguing of both potential propagules (all traded species) and known invasive alien species, can inform risk analyses and facilitate pre- or post-border control measures (i.e., import restrictions and quarantine). In circumstances where species cannot be diagnosed easily by morphology and/or only certain life history stages can be identified, standardised molecular protocols for species identification are important for biosecurity [9]–[11]. However, these techniques still require further testing and reference libraries need to be expanded to encompass more species.

The ornamental aquatic industry is among the world's largest transporters of live animals and plants, with an annual trade volume estimated at US$15–25 billion [12], [13]. Data from the United States implicates the industry as the primary transport vector in 37 of 59 fish introductions [6]. In Singapore–a global aquarium fish trading hub–at least 14 invasive ornamental fish species were reported to be resident in reservoirs in 1993 [14]. The risks presented by this industry are not, however, limited to traded invasive fishes. Associated pathogenic organisms such as protozoa, bacteria and viruses are equally undesirable, with exotic pathogens known to cause harm to native species [15], industrial food aquaculture [16]–[18], and also the ornamental fish trade [13]. Compounding this, some pathogens can be vectored by carrier hosts with no clinical signs of disease [13], [15], [18], and host-taxon specific pathogens may also require special quarantine measures [13], [18].

Aquarium fishes are both wild caught, and captive bred at aquaculture facilities, with over one billion fishes traded through more than 100 countries in 2000 [18]. In the case of freshwater fishes, 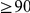
% of the trade volume is in a relatively small number of popular species sourced from commercial farms [19], while more diverse wild caught exports contribute the remainder. A complex supply chain exists for these ornamental fishes, and before they arrive at a retailer they may have passed though a series of regional and international distribution centres where consignments can be consolidated, reconsolidated and subdivided [13]. This potentially increases the number of access points for undesirable organisms to enter each shipment [13], as well as opportunities for mislabelling. While statistics are available on total volumes sold, little quantitative data exist on the number and composition of species involved in the aquarium trade, but it has been estimated that up to 5,300 species have been available at some point [20]. The industry in aquatic ornamentals for the aquarium hobby is a dynamic business, with new and undescribed species frequently appearing from new areas. Some, such as *Puntius denisonii* have quickly moved from obscurity to becoming a major Indian export and a conservation concern within a few years [21], [22].

Approaches to addressing biosecurity threats from ornamental fishes are varied; the United States and United Kingdom adopt a “blacklist”, whereby a small group of known high-risk species are subject to controls [23], [24], while countries such as Australia and New Zealand who view this industry as a greater biosecurity threat, permit only fishes included on a “whitelist” of manageable species [17],[18],[24],[25]. A total of 82 cyprinid (Teleostei: Cypriniformes: Cyprinidae) fish species are permitted for import as ornamentals in New Zealand [25]. Of these 82 species, 27 are further classified “high-risk” in terms of disease susceptibility, and require specific mitigation measures [25]. For the enforcement of these restrictions, an effective biosecurity procedure requires fast and accurate early detection of potentially harmful fishes at the pre-retail quarantine stage. For a variety of reasons, however, it may be difficult for inspectors to definitively identify all species likely to be encountered [17], [26].

Use of the standardised mitochondrial cytochrome *c* oxidase subunit I (COI) DNA barcoding protocol, *sensu* Hebert *et al.* [27], [28], has been demonstrated as an effective fish identification tool in situations including consumer protection [29]–[31] and fisheries management/conservation [32], [33]. Steinke *et al.* [34] also effectively demonstrated application of this technique for the trade in marine ornamental fish species, with their study reporting a high rate of identification success.

Here, we test this DNA barcoding approach for identification of ornamental cyprinid fishes obtained from aquarium retailers and wholesalers. Of the global diversity of 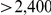 cyprinid fish species [35], some such as the barbs, danios and rasboras are popular aquarium or pond fishes, and are commonly available in petshops. Many are difficult to identify based on morphological features, and some represent risks in terms of their potential as invasive species and pathogen vectors [6], [18], [25]. We test the DNA barcoding method by comparing congruence of taxonomic identifications based on morphological features, with the patterns in DNA barcodes. In order to expand taxon coverage we also evaluate the utility of extra data from GenBank and the Barcode of Life Data System, Bold [28]. These databases will include sequences for additional species, but may also include sequences from misidentified specimens or specimens collected from otherwise unsampled, divergent populations [26], [36], [37]. Therefore, we conduct separate analyses for our own data, GenBank/Bold data, and all data combined. In addition, we use a range of different identification techniques in order to address criticisms of some commonly employed methods [37]–[41], and also incorporate a measure of how rare species affect identification success [42].

As well as testing barcodes against morphological data, nuclear loci are increasingly used to validate mitochondrial results and also provide an independent, additional source of data for both identification, systematics or taxonomy [38]. In the case of aquarium fishes, a nuclear marker may also offer advantages in detecting natural introgression patterns, or interspecific hybridisation events that may have occurred during indiscriminate or deliberate breeding at ornamental fish farms. We will assess the utility of nuclear rhodopsin (RHO), a marker having been observed to show variation at the species level for molecular systematic questions [43], and also demonstrated to serve as an effective component of a multi-locus fish identification tool [44].

With the tendency of DNA barcoding studies to discover putatively cryptic taxa [45], it is likely that our study also uncovers previously unrecognised lineages that may represent species [46]. Some researchers have even questioned the validity of cryptic taxa as reported by divergences in mtDNA analyses [47]–[49], insisting species status be additionally supported with independent datasets, *sensu* the “integration by congruence” of Padial *et al.* [50]. Nuclear markers can assist in the critical assessment of these lineage divergences, so to this effect, RHO will also be used here to test support for these hypotheses.

## Materials and Methods

### Ethics Statement

Where applicable, this study was carried out in accordance with the recommendations of the National University of Singapore Institutional Animal Care and Use Committee (IACUC) under approved IACUC protocol number B10/06 (proposal entitled “Raffles Museum of Biodiversity Research Day to Day Operations”); living fishes were kept, photographed, and handled according to these rules in the cryo-collection of the Raffles Museum of Biodiversity Research.

### Data Collection and Sampling

Specimens of ornamental cyprinid fishes were acquired from aquarium retailers, wholesalers and exporters in the United Kingdom, Singapore and New Zealand from 2008 to 2010. The non-cyprinid taxa *Gyrinocheilus* and *Myxocyprinus* were also included due to their ubiquity and superficial morphological similarity to some cyprinid fishes. Specimens were euthanised with MS-222 (tricaine methane sulfonate), before a tissue sample was excised from the right-hand caudal peduncle and stored at 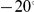
C in 100% ethanol. Specimens were subsequently formalin fixed and preserved in 70% ethanol as vouchers, following procedures outlined by Kottelat and Freyhof [51]. At least one specimen from each sample was photographed alive (left-hand side) prior to tissue sampling, with the remainder photographed after preservation. Voucher specimens for each COI barcode were deposited at the Raffles Museum of Biodiversity Research (ZRC), National University of Singapore.

Specimens were identified morphologically using scientific literature relevant to the group, and original descriptions were consulted where possible. The use of “sp.”, “cf.” and “aff.” notation in reference specimen identification follows Kottelat and Freyhof [51]. For analytical purposes, individuals designated “cf.” are treated as conspecific with taxa of the same specific name, while those designated “aff.” are treated as non-conspecific. Nomenclature follows Eschmeyer [52], unless otherwise stated. To assess the coverage of the project, a list of species believed to be in the aquarium trade was consulted as the most up-to-date and accurate guide available at this time [20]; we also used the MAF Biosecurity New Zealand Import Health Standard list of species [25].

Whenever possible, multiple individuals of each species were sampled. In order to better assess intraspecific genetic diversity, we tried to purchase multiple specimens at different times and from different vendors. Sampling efficiency was tested by correlating the number of haplotypes observed in each species with the number of individuals collected and the number of samples taken. For this purpose, a sample was considered as all conspecific specimens acquired from the same holding tank at the same premises on the same visit. These analyses were carried out in R version 2.12.1 [53], using a generalised, linear regression model with poisson distributions for count data; singleton species (species represented by one individual) were omitted.

### DNA Protocols

Approximately 2–3 mm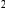 of white muscle tissue was prepared for genomic DNA extraction using the Quick-gDNA spin-column kit (Zymo Research Corporation) following the manufacturer's protocol, but scaled to use a 50% volume of pre-elution reagents. Optimised PCR reactions were carried out using a GeneAmp 9700 thermocycler (Applied Biosystems) in 10 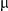
l reactions. Amplification of the COI barcode marker comprised reactions of the following reagents: 2.385 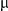
l ultrapure water; 1.0 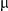
l Expand High Fidelity 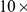 PCR buffer (Roche Diagnostics); 0.54 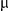
l MgCl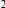 (25.0 mM); 2.0 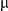
l dNTPs (1.0 mM); 1.5 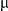
l forward and reverse primer (2.0 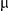
M); 1.0 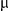
l DNA template; 0.075 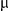
l Expand High Fidelity polymerase (Roche Diagnostics). The COI fragment was amplified using one of the following primer pairs: FishF1 and FishR1 [54], LCO1490 and HCO2198 [55], or LCO1490A and HCO2198A [56]. Thermocycler settings for COI amplification were as follows: 2 min at 94

C; 40 cycles of 15 s at 94.0

C, 30 s at 48.0–52.0

C and 45 s at 72.0

C; 7 min at 72.0

C; 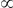 at 4.0

C.

The nuclear RHO data were generated as per the COI protocol, but using the primers RH28F [57] and RH1039R [58], and the following reagents: 1.7 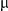
l ultrapure water; 1.0 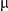
l Expand High Fidelity 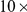 PCR buffer (Roche Diagnostics); 2.0 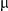
l Q-Solution (Qiagen); 0.2 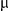
l MgCl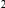 (25.0 mM); 2.0 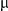
l dNTPs (1.0 mM); 1.0 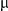
l forward and reverse primer (2.0 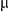
M); 1.0 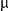
l DNA template; 0.1 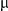
l Expand High Fidelity polymerase (Roche Diagnostics). Thermocycler settings for RHO amplification were as follows: 4 min at 94.0

C; 40 cycles of 20 s at 94.0

C, 30 s at 54.0–56.0

C and 60 s at 72.0

C; 7 min at 72.0

C; 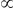 at 4.0

C.

Prior to sequencing, PCR products were checked visually for quality and length conformity on a 1% agarose gel. Bidirectional sequencing was carried out following the manufacturer's protocol on a Prism 3130xl Genetic Analyser (Applied Biosystems) using the BigDye Terminator v3.1 Cycle Sequencing Kit (Applied Biosystems). The same primer combinations as for PCR amplification were used for sequencing. Sequencing products were purified using the Agencourt CleanSEQ system (Beckman Coulter Genomics). Steps undertaken here to avoid or identify cross-amplification of nuclear mitochondrial pseudogenes (NUMTs) are outlined by Buhay [59] and Song *et al.* [60]. Sequence chromatograms were inspected visually for quality and exported using FinchTV 1.4 (Geospiza). Trimmed nucleotide sequences were aligned according to the translated vertebrate mitochondrial amino acid code in the program Mega 4.1 [61]. The resulting COI fragment comprised a sequence read length of 651 base pairs (bp), positionally homologous to nucleotides 6,476 through 7,126 of the *Danio rerio* mitochondrial genome presented by Broughton *et al.* [62]. The RHO fragment corresponded to an 858 bp length (sites 58–915) of the *Astyanax mexicanus* rhodopsin gene, GenBank accession U12328 [44], [63]. For COI and RHO, sequence data, chromatogram trace files, images and supplementary information were uploaded to Bold, and are available in the “Ornamental Cyprinidae” [RCYY] project. In addition to sequence data generated here, public databases including GenBank and Bold were searched under the following terms: “Cyprinidae”, “COI”, “CO1” and “COX1”. Records were retained if the taxon in question was believed to occur in the aquarium trade [20], or if congeneric to a species we had already collected in our sampling. To facilitate analysis, nomenclature and spellings of GenBank/Bold records were updated or corrected following Eschmeyer [52].

### Analysis

The suitability of COI barcodes as a species identification tool was tested using five primary metrics, thereby quantifying different properties of the data. Rather than simply providing a species-based descriptive summary, we simulated a real identification problem for a biosecurity official by treating each individual as an identification query. In effect, this means that each sequence is considered an unknown while the remaining sequences in the dataset constitute the DNA barcoding database that is used for identification. Identification rates for these queries were divided into four categories: “correct” or “incorrect”, and “no identification” or “ambiguous” if applicable to the method. The extent to which rare, singleton specimens (one specimen per species) affect identification success rates is rarely explored, and is a problem for DNA barcode identification systems [42]. As few taxon-specific barcoding projects (i.e., databases) are complete [42], we aim to examine how the data perform for these singletons. It is therefore important for our analyses to distinguish between two identification scenarios. First, a query specimen belongs to a species that has already been barcoded and whose DNA barcode is maintained in a DNA barcoding database. Once sequenced, the best identification result for such a specimen is a “correct identification”. Second, the query specimen belongs to a species that remains to be barcoded (it is a singleton). The best result here is “no identification”, since the specimen has no conspecific barcode match in the database. The best overall identification technique is one that maximises identification success for scenario one, and yields a “no identification” result under scenario two. In light of this, we report results with both singleton species included (scenario two) and excluded (scenario one). When the analyses were carried out, however, the singletons remained in all datasets as possible matches for non-singletons. We term the success rates for scenario one (singletons excluded) as the “re-identification rate”.

Unless otherwise stated, all descriptive statistics and analyses were conducted using Spider, Brown *et al.*'s DNA barcode analysis package for R [64], [65]. Distance matrices and neighbour-joining (NJ) phylograms were generated under Kimura's two-parameter model (K2P/K80), with missing data treated under the “pairwise deletion” option. The K2P model was only used here to ensure consistency and comparability with other barcoding studies, but see Collins *et al.* [66] and Srivathsan and Meier [67] for more general discussion on the applicability of the K2P model. Negative branch lengths were set to zero [68], [69]. Terminology of topological relationships follows phylogenetic nomenclature consistent with literature but applies only to the gene tree relationships (e.g. monophyly, paraphyly, polyphyly). NJ phylograms were rendered in Web-based jsPhyloSVG format [70], following conversion from Nexus format into phyloXML using Archaeopteryx [71]. This creates an interactive vector-graphic phylogram with links to specimen database records and supplementary data (e.g. images) via embedded URLs.

The five primary metrics measuring identification success rates in this study are described as follows: (1) We employed a tree-based test of species monophyly, with this measurement reporting the exclusivity of the genetic clusters in an NJ phylogram. The procedure returns each species as either monophyletic (correct identification), non-monophyletic (incorrect identification) or singleton (incorrect identification). This per-species measure was then scaled to include the number of individuals in each species. We also incorporated a bootstrap test of node support, with correct identifications scored if values were greater than 70% [72]; 1,000 replications and codon resample constraints (block 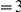 option) were used for the bootstrap analysis. (2) A test using the *k*-nearest neighbour (*k*-NN) or “best match” classification approach [37], [73] was employed on the K2P distance matrix. A nearest neighbour (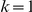) conspecific with the query returned a correct identification, otherwise an incorrect identification; singletons were reported as an incorrect identification, and ties were broken by majority, followed by random assignment. (3) We used the “best close match” (BCM) method presented by Meier *et al.* [37]. In BCM, ties are reported as ambiguous and matches must be within a pre-specified threshold value (i.e., 1%) otherwise no identification is returned [37]. (4) Fourthly, the data were tested with a technique approximating the threshold method used by the Bold-IDS identification engine [28]. Bold-IDS will return a positive identification if a query shares a 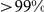 similar unambiguous match with a reference specimen [28]. Here, data were tested on a per-individual basis, using the K2P distance matrix. A correct identification was returned if all distances within 1% of the query were conspecific, an incorrect identification resulted when all distances within the threshold were different species, while an ambiguous identification result was given when multiple species, including the correct species, were present within the threshold. This method is similar to BCM, but operates upon all matches within the threshold, rather than just nearest neighbour matches.

Lastly, we used a method incorporating an estimation of group membership; the general mixed Yule-coalescent (GMYC) models the probability of transition between speciation-level (Yule model) and population-level (coalescent model) processes of lineage branching [74], [75]. This offers a likelihood based test of biological pattern in the data, i.e., approximating the “barcoding gap” of intraspecific versus interspecific variation. Following Monaghan *et al.* [75], data were reduced to haplotypes using Alter [76], with gaps treated as missing data (ambiguous bases were first transformed to gap characters). Next, ultrametric chronograms were generated in Beast v1.6.1 [77], [78] under the following settings: site models as suggested by the BIC in jModelTest [79], [80]; strict molecular clock; 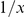 Yule tree prior; two independent MCMC chains with random starting topologies; chain length 20 million; total 20,000 trees; burn-in 10%; all other settings and priors default. The GMYC model was fitted in the Splits package for R [75], using the single threshold method under default settings. An individual was scored as a correct identification if it formed a GMYC cluster with at least one other conspecific individual. An incorrect identification was made when an individual clustered with members of other species, and a “no identification” was made when an individual formed a single entity (did not cluster with anything else). Exploratory results (data not shown) suggested that more sophisticated Beast and GMYC analyses using relaxed clocks, codon partitioned site models, outgroups, and multiple threshold GMYC resulted in a poorer fit to the morphologically identified species names, as did a full dataset (sequences not collapsed into haplotypes).

The use of a universal (e.g. 1%) threshold has been questioned repeatedly [37], [41], [81], [82], and although no single threshold is likely to suit all species, error can be minimised across a dataset for different threshold values. We tested a range of threshold percent values for their effect on both the false positive (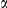) and false negative (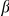) error rates. Categorisation of these error rates follows Meyer and Paulay [82]: “False positives are the identification of spurious novel taxa (splitting) within a species whose intraspecific variation extends deeper than the threshold value; false negatives are inaccurate identification (lumping) within a cluster of taxa whose interspecific divergences are shallower than the proposed value” (p. 2230). The optimum threshold is found where cumulative errors are minimised. Positive identifications were recorded when only conspecific matches were delivered within the threshold percent of the query. False negative identifications occurred when more than one species was recorded within the threshold, and a false positive was returned when there were no matches within the threshold value although conspecific species were available in the dataset. We incorporated a modification of the Bold and BCM analyses, using the revised threshold values generated during this procedure.

To evaluate the performance of the COI barcodes in terms of their agreement with nuclear RHO, a subset (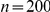) of individuals were amplified for this marker. This yielded reduced datasets of 82 species (1–10 individuals per species) for which both the COI and RHO sequences were available. Barbs (*Puntius*) and danios (Danionini) were targeted, along with other taxa showing COI divergences. Patterns in the matched RHO and COI subsets were investigated using the NJ monophyly and *k*-NN methods. When a sufficient number of specimens were available (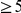) for aquarium species showing multiple COI clusters, we were able to explore this possibly unrecognised diversity with RHO, and assess an approach complementary to COI barcoding. We used four methods in assessing support for unrecognised or cryptic species: mean intergroup K2P distances; a character based approach using diagnostic, fixed character states between lineages, i.e., pure, simple “characteristic attributes” (CAs) [29], [83]; bootstrap estimates of NJ clade support (settings as described above); and Rosenberg's *P*, a statistical measure testing the probability of reciprocal monophyly over random branching processes [84].

## Results

A total of 678 cyprinid fish specimens were collected during the study, and these were identified to 172 species in 45 genera using morphological characters (refer to Table S1 for identifications, characters, taxonomic comments and bibliography). The survey of GenBank and Bold databases contributed a further 562 COI sequences from 157 species, with 81 of the species represented in both GenBank/Bold data and our data. With regard to the aquarium trade, the taxon coverage of this study represents 131 (39%) of the 333 aquarium cyprinid fishes listed in Hensen *et al.* [20], a proportion which increased to 56% coverage when GenBank/Bold data were also included. An additional 41 species not present in this inventory [20] were reported from our survey of the trade. In terms of biosecurity risk, our taxon sample covered 78% (85% including GenBank/Bold) of the 27 cyprinid fish species listed as high-risk allowable imports to New Zealand [25]; of the total 82 permitted cyprinid fishes, our data represented 79% of these (90% including GenBank/Bold).

DNA barcodes were successfully amplified from all samples in the study with the primers reported. All nucleotides translated into functional protein sequences in the correct reading frame, with no stop codons or indels observed in the data. In our COI barcode dataset, each species was represented by an average of 3.9 individuals (2.32 sampling events), with twenty species by one individual (11.6%), and 102 (59%) by 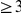 individuals. The average number of haplotypes per species was 1.97, with sampling effort (sampling events and number of individuals per sp.) and haplotype diversity correlated (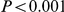). Table 1 provides a further summary of barcode statistics, and links to Bold and GenBank database records for all sequences used in this study are presented as URLs in Figure S1 and Figure S2. All sequence data used in this study are also provided as supplementary text files (Fasta format): Dataset S1 (COI) and Dataset S2 (RHO).

**Table 1:** Summary of descriptive barcode statistics for the three data partitions analysed in the study.

Statistic	This study	GenBank/Bold	Combined
Individuals	678	562	1240
Species (no. unique sp.)	172 (91)	238 (157)	329
Mean individuals per sp. (range)	3.9 (1–12)	2.4 (1–42)	3.8
Singletons	20	125	97
Genera	45	63	65
Mean sampling events per sp. (range)	2.32 (1–8)	-	-
Mean seq. length bp (range)	645 (378–651)	639 (441–651)	643 (378–651)
No. barcodes 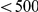 bp	5	1	6
Mean haplotypes per species	1.97 (1–7)	1.61 (1–8)	2.07 (1–10)
Mean intraspecific dist. (range)	0.90% (0–14.7%)	0.86% (0–24.1%)	1.13% (0–24.1%)
Mean smallest interspecific dist. (range)	9.11% (0–23.2%)	8.40% (0–26.0%)	8.06% (0–26.0%)
95% intraspecific var. 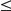	5.48%	2.13%	6.85%
95% smallest interspecific dist. 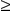	1.72%	0.00%	0.15%
Prop. intraspecific dist. 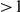 %	19.0%	32.2%	28.3%
Prop. intraspecific dist. 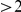 %	13.5%	5.90%	12.7%

Ranges or subsets are presented in parentheses. Abbreviations: dist. = distance(s); no. = number; prop. = proportion; seq. = sequence; sp. = species; tot. = total; var. = variation. “Combined” refers to data generated in this study combined with collected GenBank/Bold data.

**Figure 1:**
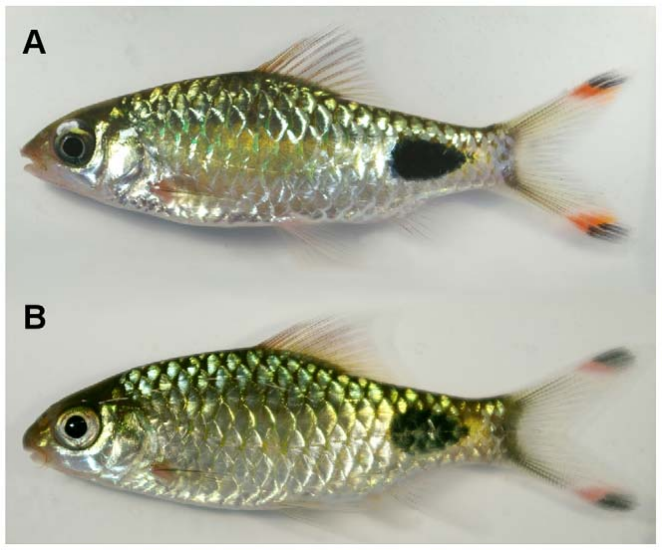
Illustrating the utility of DNA barcodes in biosecurity. *Puntius filamentosus* (A) and *P. assimilis* (B) are two species strikingly similar in appearance; morphological differences are especially difficult to discern when these are exported as juveniles. Here, we demonstrate they can be readily separated by DNA barcodes, with the two specimens pictured here differing by a 17.6% divergence in K2P distance for COI.

Genetic diversity was generally lower within species than between, with 95% of total intraspecific variation less than 5.48% K2P distance. Of the interspecific distances to a closest non-conspecific neighbour (i.e., the “smallest interspecific distance” of Meier *et al.* [85]), 95% were above 1.72% K2P distance. Mean distance to closest non-conspecific was 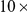 mean intraspecific distance. Of the intraspecific values, 13.5% were over 2% K2P distance, while 19.0% were above 1%. Graphical structure of the distance data is shown in the NJ phylogram presented as Figure S1, and indicates cohesive clusters for the majority of species. Many morphologically similar species were well differentiated with DNA barcodes, and Figure 1 illustrates an example.

### Identification Success Rates using DNA Barcodes

**Figure 2:**
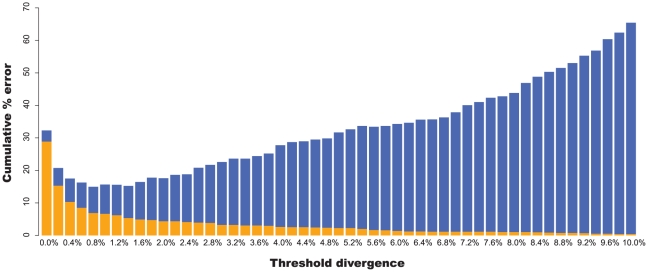
Cumulative error and threshold optimisation. False positive (orange) and false negative (blue) identification error rates summed across a range of distance thresholds from 0–10% in 0.2% increments (combined data). Definition of errors follows Meyer and Paulay [82]. Optimum threshold is 0.8%.

When appraising the identification power of the barcode data, success rates were generally high (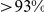) when singletons were excluded (i.e., re-identification). The only exception was the NJ bootstrap analysis (89.7%). When GenBank/Bold data were added, correct re-identification rates dropped between 4% and 15% depending on identification technique. If singleton species were included in the results, the reduction in success rate was between 2.7% and 2.9% for the data generated in this study, and 5.2% and 7.4% when GenBank/Bold data were combined. When just the GenBank/Bold data were considered, success rates decreased between 13.6% and 20.8% depending on the method. Optimised distance thresholds were 1.4% for the barcodes in this study and 0.8% when combined with GenBank/Bold (Figure 2). A breakdown of identification success rate for each method and for each dataset is presented in Table 2.

**Table 2:** Identification percent success rates for each of the five primary analytical methods across three data partitions (with singletons both included and excluded from results), plus optimum threshold values from cumulative error estimation.

Measure	Singletons	This study (%)	GenBank/Bold (%)	Combined (%)
NJ mono.	excl.	96.7 (3.3)	83.5 (16.5)	84.7 (15.3)
	incl.	93.8 (6.2)	64.9 (35.1)	78.1 (21.9)
NJ mono. boot.	excl.	89.7 (10.3)	78.7 (21.3)	74.7 (25.3)
	incl.	87.0 (13.0)	61.2 (38.8)	68.9 (31.1)
*k*-NN ( 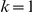 )	excl.	98.9 (1.1)	93.6 (6.4)	94.8 (5.2)
	incl.	96.0 (3.9)	72.8 (27.2)	87.4 (12.6)
GMYC	excl.	94.2 (3.6, 2.1)	72.1 (17.3, 10.5)	82.2 (12.5, 5.3)
	incl.	91.4 (3.5, 5.0)	58.5 (14.1, 27.4)	77.0 (11.7, 11.3)
Bold: 1% thresh.	excl.	93.2 (0.0, 3.2, 3.6)	75.3 (2.5, 12.8, 9.4)	82.9 (1.5, 6.6, 8.9)
	incl.	90.4 (0.0, 6.0, 3.6)	58.5 (5.3, 28.8, 7.3)	76.5 (2.8, 12.5, 8.2)
Bold: opt. thresh.	excl.	93.9 (0.0, 2.4, 3.6)	75.3 (2.5, 12.8, 9.4)	83.4 (1.7, 6.9, 8.0)
	incl.	91.2 (0.0, 5.3, 3.5)	58.5 (5.3, 28.8, 7.3)	76.9 (2.9, 12.0, 7.3)
BCM: 1% thresh.	excl.	94.8 (0.2, 3.2, 1.8)	77.6 (3.4, 12.8, 6.2)	86.7 (2.4, 6.6, 4.2)
	incl.	92.0 (0.1, 6.0, 1.8)	60.3 (6.0, 28.8, 4.8)	79.9 (3.7, 12.5, 3.9)
BCM: opt. thresh.	excl.	95.6 (0.2, 2.4, 1.8)	77.6 (3.4, 12.8, 6.2)	86.5 (2.4, 6.9, 4.2)
	incl.	92.8 (0.1, 5.3, 1.8)	60.3 (6.0, 28.8, 4.8)	79.8 (3.5, 12.9, 3.9)
Opt. thresh. value		1.4	1.0	0.8

Values in parentheses show failure rate broken down into “misidentification”, “no identification” and “ambiguous” (BCM and Bold only) respectively. “Combined” refers to data generated in this study combined with collected GenBank/Bold data. Abbreviations: BCM = “best close match”; boot. = bootstrap (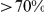); excl. = excluded; incl. = included; mono. = monophyly; opt. = optimum; thresh. = threshold.

### Incongruence between Morphology, DNA Barcodes, and GenBank/Bold Data

**Figure 3:**
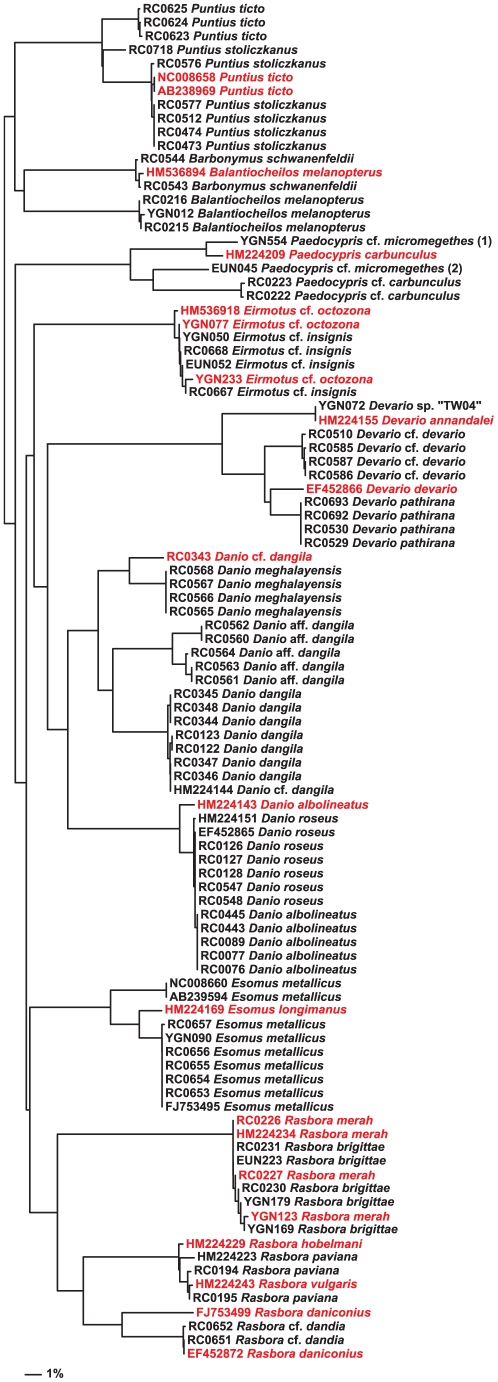
Incongruences and inconsistencies in barcode data. This reduced-taxon NJ phylogram highlights cases of haplotype sharing and paraphyly/polyphyly between nominal species. Data generated in this study are prefixed “RC0”, “YGN” and “EUN” (otherwise GenBank), with anomalous individuals represented in red.

Cases of incongruence and inconsistency for some common aquarium species are presented in a reduced NJ phylogram (Figure 3). Of the data generated in this study, barcode sharing was observed in two groups: between two *Eirmotus* species (*E*. cf. *insignis* and *E*. cf. *octozona*), and between two *Rasbora* species (*R. brigittae* and *R. merah*). Additionally, a polyphyletic species was observed: an individual of *Danio* cf. *dangila* (RC0343) clustered closer to *D. meghalayensis* than to other *D. dangila*. When GenBank data were added, several additional species were also non-monophyletic on the COI phylogram, with these added data conflicting with some barcodes generated in this study. For example, *D. albolineatus* became polyphyletic with the inclusion of *D. albolineatus* HM224143, as did *D. roseus* when *D. roseus* HM224151 was added. The topology of the NJ phylogram (Figure 3) is misleading for identification purposes, however, as all *D. roseus* remain diagnosable from *D. albolineatus* by a single transversion at position 564, while the remaining differences in *D. roseus* HM224151 are autapomorphies. Other aquarium species that were affected by GenBank data inclusion include (refer to Figure S1): haplotype sharing between a possibly undescribed *Devario* (“TW04”) and *D. annandalei* HM224155; haplotype sharing and polyphyly of *R. daniconius* and *R.* cf. *dandia*; paraphyly of *Barbonymus schwanenfeldii* by *Balantiocheilos melanopterus* HM536894; paraphyly of *Devario* cf. *devario* by *D. devario* EF452866; polyphyly of *Paedocypris carbunculus*; paraphyly of *Puntius stoliczkanus* with polyphyletic *P. ticto*; polyphyly of *R. paviana* with regard to *R. hobelmani* HM224229 and *R. vulgaris* HM224243; polyphyly of *Esomus metallicus*.

### Nuclear Data and Unrecognised Diversity

When comparing suitability of COI and RHO as a species level marker in our reduced, matched datasets, the NJ monophyly analysis yielded 98.6% success rate for COI, and 87.8% for RHO. The rates for the nearest neighbour analyses (*k*-NN) were 99.0% for COI, and 92.2% for RHO. The two genes representing two different genomes produced consistent results, but with the nuclear data performing slightly poorer at discriminating some closely related species. A NJ phylogram of RHO data is presented in Figure S2. Taxa unable to be resolved by RHO include some members of the *Puntius conchonius* group including *P. padamya*, *P. tiantian* and *P. manipurensis*. *Danio albolineatus*/*D. roseus* were also unresolved, as were *Microdevario kubotai*/*M. nana*, plus *Devario* cf. *browni* and other associated undescribed/unidentified *Devario* species. The hybrid *Puntius* clustered close to *P. arulius* in the COI NJ phylogram (Figure S1), while it clustered with *P. denisonii* in the RHO phylogram (Figure S2). This result indeed supports its identification as a hybrid, and potentially identifies the parental species.

**Figure 4:**
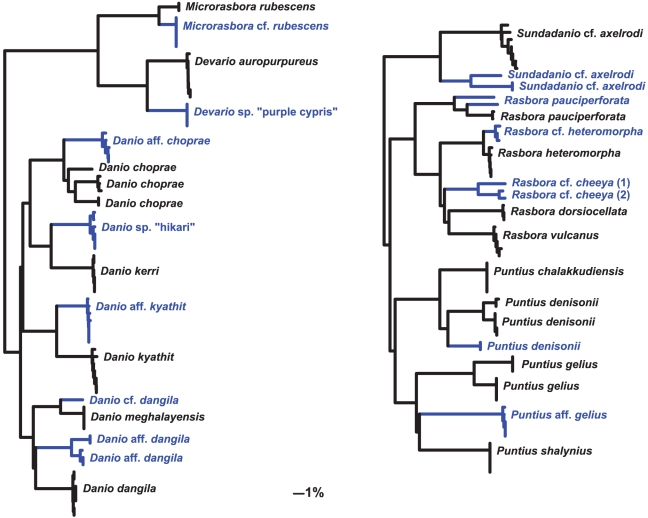
Cryptic and unrecognised species. An NJ phylogram showing deep COI barcode divergences in selected ornamental species. Taxa of interest are highlighted in blue.

In the COI data, divergent lineages (e.g. 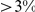) were found to be present within several common aquarium species, including: *Danio choprae, D. dangila, D. kyathit, Devario devario, Epalzeorhynchos kalopterus, Microdevario kubotai, Microrasbora rubescens, Puntius assimilis, P. denisonii, P. fasciatus, P. gelius, P. lateristriga, P. stoliczkanus, Rasbora dorsiocellata, R. einthovenii, R. heteromorpha, R. maculata, R. pauciperforata and Sundadanio axelrodi*. Some were expected, based on the morphological examination process, to be unrecognised diversity (noted by “sp.”, “cf.” or “aff.”), and some were divergent in the absence of apparent morphological differences (i.e., so-called “cryptic” species). Divergent COI lineages of species sequenced in this study are represented as an NJ phylogram in Figure 4. A numerical summary of some of these is presented in Table 3, where nuclear RHO data were used to explore whether the COI relationships were supported [48]. We find here that when COI splits were large, the RHO distances were also large, albeit on average 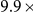 smaller (range 3.8–22.7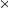). Discrete character states were observed for all species in both genes, but were again fewer at the nuclear locus and also corresponded to lower bootstrap support. Rosenberg's *P* statistic of reciprocal monophyly showed adequate sample sizes for most comparisons, but highlighted where further sampling would be beneficial.

**Table 3:** Exploring unrecognised diversity: undescribed and putative cryptic species were assessed with COI and nuclear RHO data in the context of their closest known congener or conspecifics.

Putative cryptic or unrecognised taxon	Taxon comparison	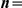	Mean K2P % COI/RHO	No. CAs COI/RHO	Bootstrap % COI/RHO	Rosenberg's *P* COI/RHO
*Danio* aff. *choprae*	*D. choprae*	6	7.4/0.5	23/2	100/92.7*	Y/N*
*Danio* aff. *dangila*	*D. dangila*	7	9.0/1.3	21/10	100/89.9	Y/Y
*Danio* aff. *kyathit*	*D. kyathit*	6	7.0/1.1	40/7	100/100	Y/Y
*Danio* sp. “hikari”	*D*. cf. *kerri*	6	8.6/0.6	48/5	100/97.1	Y/Y
*Devario* sp. “purple cypris”	*D. auropurpureus*	6	8.1/0.6	47/5	100/99.8	Y/Y
*Microrasbora* cf. *rubescens*	*M. rubescens*	5	3.7/0.5	23/3	100/95.3	N/N
*Puntius* aff. *gelius*	*P. gelius*	7	17.2/4.1	76/27	100/100	Y/Y
*Puntius denisonii*	intraspecific	5	7.8/0.4	40/3	100/95.7	N 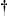 /N
*Rasbora* aff. *dorsiocellata* 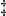	*R. dorsiocellata*	6	10.9/1.5	46/8	100/82.5	Y/Y
*Rasbora* cf. *heteromorpha*	*R. heteromorpha*	7	2.2/0.2	11/1	100/18.1	Y/N
*Sundadanio* cf. *axelrodi*	intraspecific	10	13.8/2.3	42/9	100/99.6	Y/Y

Notes: (*) renders *Danio choprae* paraphyletic; (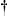) *P* monophyly significant to the 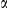 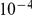 level with combined COI data (15 specimens); (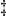) species likely described during manuscript preparation as *Brevibora cheeya* [99]. Abbreviations: CA = pure, simple characteristic attribute (i.e., discrete diagnostic character state); Y = Rosenberg's *P*, significant to 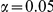; N = not significant.

## Discussion

### Sampling

Accurately assigning correct taxonomic names to voucher specimens and barcodes is a critical first step in assembling a useful reference library for non-expert users. Unlike previous studies of regional faunas [86], [87], scientific publications covering all taxa likely to be encountered in the aquarium trade were not available. In some cases, reliable guides to local faunas and up-to-date revisions existed, but in other cases such as Indian fishes, little taxonomic research has been conducted since the original descriptions from the early 19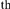 century. Liberal use of the “cf.” notation where specimens examined differed from diagnoses in the literature (29 examples), is testament to the uncertainty in identification based on these data.

Our survey of the trade revealed that 24% of species available were not listed in the most recent and thorough reference list for the trade [20], indicating a mismatch between actual availability and published literature. Conversely, many species listed in this reference did not appear to be available at the wholesalers and retailers visited. Some of these discrepancies surely arise from identification and nomenclatural issues, but is otherwise likely due to changing export patterns through different regions and time.

A strong relationship between haplotype diversity and sample frequency was observed, indicating that expanding the reference library will result in the discovery of further genetic variability. In terms of the patterns of trade, we predict that farmed species will have a lower genetic diversity and fewer observed haplotypes than those of wild caught species, which may make them easier to identify with DNA barcodes. Preliminary investigations have suggested that this may well be the case, but due to difficulties obtaining reliable information through the supply chain and problems with establishing independence of samples (i.e., “independent” samples may have derived from a single source), these observations should be investigated further.

### Identification Success Rates using DNA barcodes

For biosecurity applications, relying upon the names provided by aquarium fish suppliers is likely to be highly inaccurate, and DNA barcoding represents a defensible approach. When we compared our morphological identifications to trade names or names in popular references used by the trade [88], we estimate that up to 25% of cyprinid species could be mislabelled. The DNA barcode library generated in this study provides an ideal tool to test this preliminary observation in more detail and provide a future quantified study of supplier mislabelling in the ornamental industry.

A particular challenge to biosecurity is the steady change in the number and identity of species that are traded. Any useful identification method must be robust to these changes; i.e., sequences from new species in the trade should not be erroneously matched to species with barcodes in the database, while a good identification technique should allow for the re-identification of species that are already represented. We do not present a full assessment of all identification methodologies, but we can here discuss the advantages and disadvantages of the methods covered in our study.

Many barcoding studies employ terminology describing, for example, species forming “cohesive clusters” differentiated from one another by greater interspecific than intraspecific divergence, i.e., the barcode gap of Meyer and Paulay [82]. In our study, we measured clustering in terms of monophyly in NJ phylograms, a tree-based method which performed well on data generated here, but suffered when combined with GenBank/Bold information. This method requires strict monophyly of each species, resulting in a situation where the inclusion of a single misidentified specimen renders all queries in that species as misidentifications. Although alternative tree-based measures are available (e.g. Ross *et al.* [39]), the use of NJ trees in general is questionable due their method of construction [29], [37] and topological uncertainty [37], [89]. Furthermore, for a variety of reasons, “good species” may not always be monophyletic at mtDNA loci, so this method may fail to recognise species with either a history of introgression, or young species with large effective population sizes retaining ancestral polymorphisms [49], [73], [90]. These problems are not resolved through the use of bootstrap values, as we observed a significant reduction in identification success rate when node support was considered (up to 10%); recently divergent sister species on short branches were often not supported, even if they were monophyletic and diagnosable. DNA barcoding aims to maximise congruence between morphological identifications and sequence information while minimising misdiagnosis, but this is seriously undermined when bootstrap support values are included. For the reasons stated above, NJ trees are best avoided as a sole identification method [91], but can be a useful way to visualise and summarise patterns within barcode data.

The BCM and *k*-NN methods do not require reciprocal monophyly of each species, but merely that the nearest neighbour (single closest match) is conspecific. Thus, even when conflicting GenBank/Bold data were included, identification success could still remain high. In cases of a tied closest match, the *k*-NN method ignores this uncertainty and will offer an identification based on majority, while the BCM method reports this as ambiguous. Similarly to NJ, practical difficulties can occur with *k*-NN when identifying a divergent query from an unsampled species or population, as there is no option for a “no identification”. This is a serious problem for undersampled datasets, but the BCM and Bold are able to offer a “no identification” result by incorporating a heuristic measure of species membership (a threshold of 1% distance divergence). Despite fundamental criticisms of threshold methods (e.g. variable molecular clock rates between lineages [92]), it at least provides an approximate criterion for separating intraspecific from interspecific variation [91]. In assessing whether the threshold of 1% best-fitted data generated in this study, the analysis of cumulative error demonstrated that error was variable depending on the dataset. However, it did not grossly depart from Bold's 1% threshold, perhaps justifying the use of this metric at least in the cases presented here. When we modified the Bold and BCM methods to employ these revised thresholds, we found slight improvements in the identification success rates. Using the Bold method of identification, all matches within the threshold need to belong to conspecifics, rather than the single closest match (as in BCM and *k*-NN). So like NJ monophyly, the Bold technique is also confounded by even a single misidentified or haplotype sharing specimen in that cluster, and will return an ambiguous result in this situation. This is advantageous when all sources of uncertainty need to be considered, but can lower the number of successful identifications. As a biosecurity tool, it is worth noting that while the method used by Bold performed well, identification rates can be improved further by adopting a method such as BCM with a revised, data-derived threshold.

The GMYC is another method incorporating a measure of species membership (a “no identification”), but rather than an arbitrary or generalised cut-off, GMYC employs biological model specification, speciation patterns and coalescent theory in estimating species-like units. As a likelihood based approach, measures of probability and support can be incorporated. Results were highly congruent with the threshold analyses, suggesting the GMYC is picking up the same signal, but optimising the method for all situations may take prior experience or significant trial and error. Another drawback is that the GMYC is not a particularly user friendly technique, requiring many steps and intensive computation, perhaps precluding its use in some border biosecurity applications where fast identifications may be required [9]. Our analysis of 663 haplotypes took approximately five days on a dual processor desktop PC, and although unquantified here, the method also appears sensitive to initial tree-building methodologies.

We reported results with both singleton species included and excluded (Table 2). The exclusion of singletons represents a re-identification scenario where a barcode database is complete and no new species are to be encountered. However, this is an unrealistic assumption here, as the traded cyprinid fishes come from a much larger pool of these fishes not currently available in the trade, and the number of singletons in our trade survey shows that it is likely that more singletons will be encountered in the future. These singleton species were usually rare/expensive species, contaminants, or bycatch. When singletons comprised a large proportion of the reference database (such as with the GenBank/Bold data), the correct identification rates were significantly reduced for all methods, but GMYC, Bold, and BCM were able to discriminate when a specimen could not be assigned to species. In this respect, the NJ and *k*-NN methods are poorly performing because they are not sensitive to the presence of singletons in a data set; they will always misidentify a query when a match is not available in the database, and this problem may preclude their use until reference databases are complete.

### Incongruence between Morphology, DNA Barcodes, and GenBank/Bold Data

Although few in number, cases of incongruence between barcodes require careful interpretation, especially where the inclusion of GenBank or Bold data result in some common aquarium species becoming ambiguous to distinguish. However, with some background knowledge inferences can be made, and incongruence falls broadly into two categories: taxonomic uncertainty, and conflict due to misidentifications. In the example of barcode sharing in *Eirmotus*, despite good quality specimens and the availability of a thorough, modern revision of the genus [93], our morphological identifications were uncertain (Table S1). DNA barcodes from this cluster could belong to either *E. octozona* or *E. insignis*, which is likely the result of these taxonomic/identification problems. Topotypic specimens would be required for a better understanding of the problem. Likewise in the case of *Rasbora brigittae* and *R. merah*, individuals of both species were observed to be inconsistent in diagnostic morphological character states (Table S1). Again, specimens clustering in this group could belong to either species, a finding which certainly warrants further taxonomic investigation. Haplotype sharing between the possibly undescribed *Devario* sp. “TW04” and GenBank *D. annandalei* is likely explained also by uncertainty in our identification of this individual, or the misidentification of the GenBank specimen. Due to the large number of undescribed *Devario* species in Asia, and few modern treatments, identification of many wild caught *Devario* is difficult. The aberrant specimen of *Danio dangila* (RC0343) displayed slight morphological differences to the other *D. dangila*, but with only one individual available, it was conservatively regarded as conspecific (Table S1). A similar observation was made with *Devario* cf. *devario* having divergent barcodes from GenBank *D. devario*, and an inconsistent morphology to that of the published *D. devario* literature. The example of *Danio albolineatus* and *D. roseus* shows a situation where all specimens from the trade are homogeneous and diagnosable, but rendered polyphyletic when data are included from other GenBank populations. This finding is perhaps expected given *D. albolineatus* (*sensu lato*) is a variable species with three synonyms, distributed across much of Southeast Asia [94].

Some examples certainly represent cases of misidentification, with specimens of GenBank “*Puntius ticto*” from the Mekong, grouping closer to *P. stoliczkanus*, a species with which it is often confused [95]. Other examples such as the paraphyly of *Barbonymus schwanenfeldii* by a GenBank *Balantiocheilos melanopterus* individual (HM536894), is probably a case of human error and poor quality control of data, given the marked morphological differences between the two species. Identifications made prior to recently published taxonomic works may also be subject to error, which may explain GenBank's sequences of *Rasbora daniconius*, a species formerly considered to be widely distributed, but now likely restricted to the Ganges drainage of northern India [96].

So should GenBank data be included in “real life” biosecurity situations? GenBank certainly offers a formidable resource in terms of taxon coverage and extra information, providing sometimes expert-identified wild-caught specimens with published locality data. However, the absence in many cases of preserved vouchers and justified identifications in GenBank undermines its utility for identification purposes [26], [36], [37]. Bold data are certainly better curated, and with higher quality standards, but are also likely to suffer from misidentified specimens to some degree [37]. Our results do show a decrease in identification success when GenBank data were used, and this was generally due to the higher proportion of singleton species and misidentified specimens, rather than conflicting genetic data *per se*. Realistically though, as long as the practitioner is aware of alternative explanations for patterns, and is also aware of the relative disadvantages with each analytical technique, there is every reason for incorporating these additional data, especially when a smaller dataset is unable to provide a match. No database is immune to errors, but in this study identifications are transparent, and characters, photographs and preserved vouchers can be scrutinised and updated at any time via Bold.

### Nuclear Data and Unrecognised Diversity

In terms of corroborating COI and assessing the suitability of a nuclear locus as a species identification tool, the RHO marker was found to be broadly consistent with mitochondrial COI and morphology. Although failing to distinguish a small number of closely related species, RHO served as a useful indicator of interspecific hybridisation in one case (*Puntius* spp. hybrid).

In terms of unrecognised diversity, significant within-species COI diversity was observed in several common ornamental species, and cases of otherwise unreported morphological variation was also recognised. For an exemplar group of aquarium species, and where sufficient numbers of individuals were available, additional support for these divergent COI lineages was assessed with the nuclear RHO marker using character-based analyses, successfully demonstrating evidence in both genomes. Implications for conservation and sustainable management of fisheries are also apparent here; we find *Puntius denisonii*–a species at risk of over-exploitation [21]–may comprise at least two possibly morphologically cryptic lineages. Although sample sizes were relatively small, these findings certainly warrant further investigation into species limits of these particular taxa. Supporting methods using nuclear data attempt to build on the solely mitochondrial approach by providing congruence with an external dataset [47]–[49]. This process provides useful reference points, therefore generating further taxonomic questions for closer examination.

### Conclusions

Despite the challenge of getting accurate identifications for many species, we have assembled a large database of demonstrably identified fishes and associated barcodes. We believe that DNA barcoding represents a significant move forward in providing identification tools for aquarium species in biosecurity situations. For the small number of cases where barcodes fail to offer unambiguous identifications, additional data such as Web-based images of live specimens, morphological characters, and nuclear loci can be called upon to resolve these problematic specimens. Benefits from barcoding extend beyond a simple quarantine tool, and provide a basis for the generation of accurate and consistent trade statistics, allowing auditing, record keeping and harmonisation between jurisdictions and agencies [97]. Benefits within the ornamental fish industry are also apparent, with accurately identified livestock providing a value added product suitable for export in compliance with international certification or legal standards [13]. Any country vulnerable to aquatic invasions of ornamental species can benefit, with barcode databases offering free and instant access to information. Additional benefits to conservation efforts arise in documenting the ornamental pet trade, with examples such as stock management, traceability, and effective regulation/enforcement of endangered and Cites controlled species [34]. Development of operational databases rely on solid taxonomic foundations [50], [82], [98], and studies such as these support taxonomy in generating new ideas as well as adding a suite of fine-scale characters and lab protocols, easily accessible via the Web.

## Supporting Information

Figure S1 NJ phylogram (COI data) of all specimens (this study plus GenBank/Bold data), in phyloXML SVG (scalable vector graphic) format. Archived version of Figure S1 may require open-source archiving software such as “7-Zip” to unpack. The interactive Web version can be found at http://goo.gl/avNuz. Data including identifiers, sequences, trace files, museum voucher codes and specimen images are accessed via the Bold and GenBank Web sites using URLs embedded in the taxon names. This figure is best viewed with Mozilla Firefox to fully enjoy the benefits of SVG and URL linking. May take up to one minute to load. A scripting “error” may appear in some browsers–this is the browser taking time to render the complex diagram. Phylogram can be saved as a pdf by printing to file using a custom paper size (approximately 3,600 mm height). Links can be opened in a new tab using Ctrl+LeftClick.
(BZ2)

Figure S2 NJ phylogram (reduced RHO data) generated in phyloXML SVG (scalable vector graphic) format. Archived version of Figure S2 may require open-source archiving software such as “7-Zip” to unpack. The interactive Web version can be found at http://goo.gl/h9sY5. Data including identifiers, sequences, trace files, museum voucher codes and specimen images are accessed via the Bold and GenBank Web sites using URLs embedded in the taxon names. This figure is best viewed with Mozilla Firefox to fully enjoy the benefits of SVG and URL linking. May take up to one minute to load. A scripting “error” may appear in some browsers–this is the browser taking time to render the complex diagram. The phylogram can be saved as a pdf by printing to file using a custom paper size (approximately 750 mm height). Links can be opened in a new tab using Ctrl+LeftClick.
(BZ2)

Table S1 Full list of specimens, identifications, morphological characters, comments, and bibliography of samples generated in this study.
(PDF)

Dataset S1 Text file containing all COI sequences used in the study (Fasta format).
(TXT)

Dataset S2 Text file containing all RHO sequences used in the study (Fasta format).
(TXT)
