# Supplementary figures and images for: Barcoding and Border Biosecurity: Identifying Cyprinid Fishes in the Aquarium Trade
Source: PLoS One. 2012 Jan 20;7(1):e28381. doi: 10.1371/journal.pone.0028381 (PMC3262790; doi:10.1371/journal.pone.0028381)

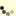

Supplement: Figure S1 — NJ phylogram (COI data) of all specimens (this study plus GenBank/Bold data), in phyloXML SVG (scalable vector graphic) format. Archived version of Figure S1 may require open-source archiving software such as “7-Zip” to unpack. The interactive Web version can be found at http://goo.gl/avNuz. Data including identifiers, sequences, trace files, museum voucher codes and specimen images are accessed via the Bold and GenBank Web sites using URLs embedded in the taxon names. This figure is best viewed with Mozilla Firefox to fully enjoy the benefits of SVG and URL linking. May take up to one minute to load. A scripting “error” may appear in some browsers–this is the browser taking time to render the complex diagram. Phylogram can be saved as a pdf by printing to file using a custom paper size (approximately 3,600 mm height). Links can be opened in a new tab using Ctrl+LeftClick. (BZ2) [file pone.0028381.s001.bz2 › gif/ajax-loader.gif]
