# Supplementary material for: Barcoding and Border Biosecurity: Identifying Cyprinid Fishes in the Aquarium Trade
Source: PLoS One. 2012 Jan 20;7(1):e28381. doi: 10.1371/journal.pone.0028381 (PMC3262790; doi:10.1371/journal.pone.0028381)
Supplement: Figure S2 — NJ phylogram (reduced RHO data) generated in phyloXML SVG (scalable vector graphic) format. Archived version of Figure S2 may require open-source archiving software such as “7-Zip” to unpack. The interactive Web version can be found at http://goo.gl/h9sY5. Data including identifiers, sequences, trace files, museum voucher codes and specimen images are accessed via the Bold and GenBank Web sites using URLs embedded in the taxon names. This figure is best viewed with Mozilla Firefox to fully enjoy the benefits of SVG and URL linking. May take up to one minute to load. A scripting “error” may appear in some browsers–this is the browser taking time to render the complex diagram. The phylogram can be saved as a pdf by printing to file using a custom paper size (approximately 750 mm height). Links can be opened in a new tab using Ctrl+LeftClick. (BZ2) [file pone.0028381.s002.bz2 › RHO.html]

Figure S2

**Figure S2 (archive version).** NJ phylogram (reduced RHO data) generated in phyloXML SVG (scalable vector graphic) format. Data including identifiers, sequences, trace files, museum voucher codes and specimen images are accessed via the BOLD and GenBank Web sites using URLs embedded in the taxon names. This figure is best viewed with Mozilla Firefox to fully enjoy the benefits of SVG and URL linking. May take up to one minute to load. A scripting "error" may appear in some browsers—this is the browser taking time to render the complex diagram. Phylogram can be saved as a pdf by printing to file using a custom paper size (approximately 750 mm height). Links can be opened in a new tab using Ctrl+LeftClick. Online version found at goo.gl/h9sY5.   


---

Loading tree. This may take up to one minute.
