# Supplementary material for: Barcoding and Border Biosecurity: Identifying Cyprinid Fishes in the Aquarium Trade
Source: PLoS One. 2012 Jan 20;7(1):e28381. doi: 10.1371/journal.pone.0028381 (PMC3262790; doi:10.1371/journal.pone.0028381)
Supplement: Table S1 — Full list of specimens, identifications, morphological characters, comments, and bibliography of samples generated in this study. (PDF) [file pone.0028381.s003.pdf]

Table S1. Characters, bibliography and specimens for morphological identifications.

Identification	Characters	Citations	Comments	Specimens
Balantiocheilos melanopterus (Bleeker)	Barbels absent; snout pointed; last unbranched dorsal ray serrated; lower lip extends posteriorly to form pocket; pelvic, anal, caudal and dorsal with wide black margins (>50% in pelvic and anal); body silver (life).	Kottelat (2001); Ng and Kottelat (2007).		RC0215 RC0216 YGN012
Barboides gracilis Brüning	Barbels absent; lateral line absent; visible humeral organ; one pair figure-8 shaped nostrils; dorsal origin anterior to pelvics; prominent axial streak; large eye (approx. 45% HL); 6½ dorsal branched rays; 5½ branched anal rays; scattered melanophores on flanks; black spot on caudal base; orange/red body colour (life).	Conway and Moritz (2006).		RC0628 RC0629
Barbonymus altus (Günther)	Two pairs barbels; short snout; last unbranched dorsal ray strongly serrated; lateral line complete (31–32 pored scales); 7½ scales between dorsal origin and lateral line; dark pigments at base of scales; caudal lobes lacking distinct black submarginal stripe; red colour to pelvics and caudal (life).	Gante et al. (2008); Kottelat (2001).		RC0178 RC0179
Barbonymus schwanenfeldii (Bleeker)	As *B. altus*, but: lateral line with 33–34 pored scales; distinct black submarginal stripe to caudal lobes.	Gante et al. (2008); Kottelat (2001).		RC0543 RC0544
Barbus callipterus Boulenger	Two pairs barbels; mouth subterminal; last unbranched dorsal ray not serrated; lateral line complete (23+2 pored scales); dorsal concave with 8½ branched dorsal rays; 5½ branched anal rays; scales with dark bases; dorsal orange anteriorly (life) with black median spot; caudal orange at base; no markings in other fins.	Boulenger (1907).	Description brief, but best match available. Boulenger (1907) reports a terminal mouth. Rows of cephalic papillae noted.	RC0613
Barbus fasciolatus (Günther)	Two pairs barbels (maxillary length = eye diameter); body slender; lateral line complete (25–30 pored scales); 8½ branched dorsal rays; 5½ branched anal rays; approx. 10–15 black vertical bars, last forming spot on caudal peduncle; spot at anal origin.	Günther (1868); Skelton (2001).		RC0035 RC0036
Barbus trispilos (Bleeker)	Two pairs barbels (rostral as long as eye diameter, maxillary approx. 1.5× eye diameter); mouth subterminal; last unbranched dorsal ray not serrated; lateral line complete, curving ventrally (24–25+2 pored scales); dorsal slightly concave with 8½ branched dorsal rays; 5½ branched anal rays; scales with dark bases; 3 distinct midlateral blotches (second and third slightly elongate).	Günther (1868); Hopson (1965).	Slightly lower lateral line scale count and shorter barbel length than reported by Hopson (1965). Rows of cephalic papillae noted.	RC0606 RC0607
Chela dadyburjori (Menon)	Barbels absent; lateral line incomplete (up to 4 pored scales); supraorbital groove present; dorsal origin posterior to that of anal; 7½ branched dorsal rays; 11½–12½ branched anal rays; elongated pectoral fins; dark midlateral stripe ending at caudal base, with 3–4 indistinct superimposed spots; no markings on fins.	Fang (2003); Menon (1952); Pethiyagoda et al. (2008).	Spelling of specific name follows Pethiyagoda et al. (2008). Generic assignment follows Tang et al. (2010).	RC0333 RC0334 RC0335 RC0336 RC0337
Crossocheilus cf. *atrilimes* Kottelat	Two pairs barbels (maxillary rudimentary or absent in larger specimens); rostral cap fimbriated; free rostral lobe absent; lower lip papillose; 8½ branched dorsal rays; approx. 1–1½ scales between anus and anal fin; black midlateral stripe extending to end of median caudal rays; fins with no distinct markings; no distinct black marking between anus and anal fin; two rows of dark dots below midlateral stripe (absent in small specimens); proximal yellow colour to fins in large specimens.	Kottelat (2000); Kottelat and Widjanarti (2005); Tan and Kottelat (2009).	Identification tentative, as inconsistency among specimens in some characters (e.g. barbels and markings).	RC0327 RC0521 RC0713 YGN232
Crossocheilus langei Bleeker	Two pairs barbels (maxillary rudimentary in larger specimens); rostral cap fimbriated; free rostral lobe absent; lower lip papillose; 8½ branched dorsal rays; approx. 2–2½ scales between anus and anal fin; black midlateral stripe extending to end of median caudal rays; fins with no distinct markings; distinct black marking between anus and anal fin.	Kottelat (2000); Kottelat and Widjanarti (2005); Tan and Kottelat (2009).	Maxillary barbels reduced/absent in RC0737: treated as *C.* cf. *langei*.	RC0287 RC0288 RC0714 RC0715 RC0737 EUN115
Crossocheilus nigriloba Popta	Two pairs barbels; rostral cap fimbriated; free rostral lobe absent; lower lip papillose; 8½ branched dorsal rays; midlateral black stripe continuing onto lower caudal lobe; red marginal stripes and tips to caudal (life).	Kottelat et al. (1993); Rainboth (1996); Roberts (1989).		RC0735 RC0736
Crossocheilus reticulatus (Fowler)	Two pairs barbels (maxillary rudimentary or absent in larger individuals); rostral cap fimbriated; free rostral lobe absent; lower lip papillose; 8½ branched dorsal rays; large dark blotch on caudal base; dark scale margins: reticulate pattern; no distinct markings in fins.	Banarescu (1986); Fowler (1934, 1935); Kottelat (2001); Rainboth (1996); Roberts (1989).		RC0388 RC0517
Cyclocheilichthys janthochir (Bleeker)	One pair barbels (minute); lateral line complete; pores on head forming dense parallel rows; black midlateral stripe; dorsal red with black anterior margin (life); caudal red with black marginal stripe (life).	Kottelat et al. (1993); Roberts (1989).		RC0614 RC0615 YGN291
Cyprinella lutrensis (Baird & Girard)	Barbels absent; lateral line complete (33 pored scales); 8½ branched anal rays; well developed tubercles on head; metallic blue body (life); dark bar behind operculum; pectoral, pelvic and caudal red (life); dorsal surface of head red (life); body with reticulate scale pattern.	Boschung and Mayden (2004); Matthews (1987).	Large number of synonyms in this species.	RC0207 RC0208

Cyprinus carpio Linnaeus	Two pairs barbels; lateral line complete (31 +1 pored scales); long concave dorsal; caudal deeply emarginate; last unbranched anal ray spinous and serrated posteriorly.	Kottelat and Freyhof (2007).	The ornamental "koi" variety is hypothesised to belong to *Cyprinus rubrofuscus* Lacepède by Kottelat and Freyhof (2007). Wild *C. rubrofuscus* should have 29–33 pored lateral line scales and this specimen agrees with the diagnosis, but due to support from a single character, and the selective breeding in ornamental varieties, the "koi" is retained here for now as *C. carpio*.	EUN226
Danio aesculapii Kullander & Fang	Two pairs barbels (rostral not extending past pectoral base); 6½ branched dorsal rays; lateral line incomplete; approx. 6 short lateral bars anteriorly, continuing into parallel rows of spots/dots; distinct A-stripe.	Kullander and Fang (2009a).		RC0111<br>RC0112<br>RC0706<br>RC0707<br>RC0708
Danio albolineatus (Blyth)	Two pairs long barbels (rostral extending to eye); lateral line incomplete (up to 9 pored scales); 7½ branched dorsal rays; body devoid of stripes except a dark P-stripe posterior on body, bordered above by light I-stripe, ending on caudal base; blue/pink colouration in life.	Fang and Kottelat (1999, 2000).	The *D. albolineatus* complex is poorly characterised and requires systematic attention. Numerous synonyms exist, but these specimens are regarded by the oldest available name.	RC0076<br>RC0077<br>RC0089<br>RC0443<br>RC0445
Danio choprae Hora	Two pairs barbels (rostral not extending past eye, maxillary not extending past pectoral base); 7½ branched dorsal rays; lateral line absent; 6–8 short lateral bars anteriorly, continuing into rows of spots and P-stripe on caudal peduncle; P+1 and P–1 stripes continue onto caudal; distinct A and D stripes.	Hora (1928); Kullander and Fang (2009a).	Spelling of specific name follows Kullander and Fang (2009a).	RC0059<br>RC0060<br>RC0079<br>RC0163<br>RC0164<br>RC0446
Danio aff. *choprae* Hora	As *D. choprae*, but barbels longer (rostral extending past eye, maxillary extending past pectoral base); lateral line incomplete (1–3 pored scales); anterior lateral bars broken up with intermediate spots; larger size; overall grey rather than orange colouration (life).	Hora (1928); Kullander and Fang (2009a).	Likely an undescribed species, differing in several characters from *D. choprae*. Spelling of specific name follows Kullander and Fang (2009a).	RC0523<br>RC0524<br>RC0525<br>RC0669<br>RC0670
Danio dangila (Hamilton)	Two pairs long barbels (maxillary reach past operculum); supraorbital groove absent; lateral line complete (32–36 pored scales); 9½–11½ branched dorsal rays; 15½ branched anal rays; well defined vertically elongated cleithral spot; network of P-stripes (blue in life) and interspaces forming spots and rings; P-stripes continue onto caudal; anal with 2–3 A-stripes.	Day (1875); Hamilton (1822); Sen and Dey (1985); Talwar and Jhingran (1991).	RC0343 appears different, with darker pattern, larger size; wider P-stripes, smaller interspace spots, a distinct axial streak, and a cleithral spot not elongated vertically. This specimen is regarded here as *Danio* cf. *dangila*.	RC0122<br>RC0123<br>RC0343<br>RC0344<br>RC0345<br>RC0346<br>RC0347<br>RC0348
Danio aff. *dangila* (Hamilton)	As *D. dangila*, but with stripes on dorsal and caudal forming distinct and discreet spots.	Day (1875); Hamilton (1822); Sen and Dey (1985); Talwar and Jhingran (1991).	Likely an undescribed *Danio* closely related to *D. dangila*. Purportedly sourced from Myanmar.	RC0560<br>RC0561<br>RC0562<br>RC0563<br>RC0564
Danio erythromicron (Annandale)	Barbels absent; lateral line absent; 7½ branched dorsal rays; supraorbital groove absent; snout short and blunt; well defined dark spot at caudal base; fins without stripes; up to 12 narrow lateral bars, from operculum to caudal peduncle.	Annandale (1918); Conway et al. (2008).		RC0552<br>RC0553<br>RC0599<br>RC0704<br>RC0705<br>YGN172<br>YGN340

Danio feegradei Hora	Two pairs long barbels (maxillary extends past operculum); lateral line complete (approx. 36 pored scales); 8½ branched dorsal rays; 12½ branched anal rays; cleithral spot present; dark P-stripe narrowing posteriorly and terminating in spot on caudal base, with light I-stripe above posteriorly (on caudal peduncle and base); light spots in two rows anteriorly.	Hora (1937).		RC0245 RC0246 RC0247 RC0248 RC0249
Danio cf. *kerri* Smith	Two pairs barbels (rostral extends past eye, maxillary beyond pectoral base); 7½ branched dorsal rays; lateral line incomplete (up to 9 pored scales); two complete lateral stripes (P and P+1) with two light interspaces, widening posteriorly and joining in a loop behind the operculum; fins dusky with weak pigmentation.	Smith (1931).	Smith (1931) reports no pored lateral line scales in *D. kerri*, so this material is regarded as *D.* cf. *kerri*.	EUN035 RC0267 RC0268 RC0269 RC0270 RC0271
Danio kyathit Fang	Two pairs long barbels (maxillary extends past operculum); supraorbital groove absent; lateral line incomplete (5–9 pored scales); 13½–14½ branched anal rays; D-stripe and 3 A-stripes present; 5–7 P-stripes broken almost entirely into spots; P, P+1 and P–1 extending onto caudal; caudal without stripes on lobes.	Fang (1998); Kullander et al. (2009).	Conforms to holotype of *D. kyathit* Fang (1998).	RC0064 RC0090 RC0129 RC0130 RC0131 YGN014 YGN338
Danio aff. *kyathit* Fang	As *D. kyathit*, but: P-stripes as stripes rather than spots; P–1 and P–2 stripes slightly ventrally slanting.	Fang (1998); Kullander et al. (2009).	A likely undescribed species with distinct colour pattern from *D. kyathit* s.s. holotype (Fang, 1998). A paratype of *D. kyathit* from Kamaing (Ayeyarwaddy drainage) shows a similar pattern. Similar also to *D. quagga* Kullander, Liao & Fang, but barbels appear longer here, and *D. quagga* is a poorly known species.	EUN041 EUN179 RC0065 RC0066 RC0120 RC0121 RC0405
Danio margaritatus (Roberts)	Barbels absent; lateral line absent; 7½ branched dorsal rays; supraorbital groove absent; snout short and blunt; D-stripe, A-stripe and A-1 stripe present; P+1 and P–1 stripes extend onto caudal; 5–6 irregular rows of spots; distinctive blue, red, gold colouration (life).	Conway et al. (2008); Roberts (2007).		RC0032 RC0033 RC0107 RC0138 RC0139
Danio meghalayensis Sen & Dey	Two pairs barbels (maxillary not reaching past operculum, rostral just extending past eye); supraorbital groove absent; lateral line complete (33–34 pored scales); 8½ branched dorsal rays; 10½–11½ branched anal rays; no distinct cleithral spot; 5 P-stripes, with interspaces forming broken golden (life) spots and stripes anteriorly; P-stripes continue onto caudal; anal with A-stripes.	Day (1875); Hamilton (1822); Sen and Dey (1985); Talwar and Jhingran (1991).		RC0565 RC0566 RC0567 RC0568
Danio nigrofasciatus (Day)	One pair barbels (maxillary, reaching past eye); P and P+1 stripes uniform unbroken, extending into caudal; no stripe above P+1; stripes below P broken into spots; anal and pelvics spotted; D-stripe present.	Fang (1998); Kullander and Fang (2009b).		EUN034 RC0081 RC0082 RC0242 RC0243 RC0244
Danio rerio (Hamilton)	Two pairs long barbels (maxillary extends past operculum, rostral not extending past eye); lateral line absent, except in RC0679 (4 pored scales); D-stripe and 3 A-stripes present; 5 well defined parallel P-stripes, with P, P+1 and P–1 extending onto caudal; caudal with stripes on lobes.	Fang (1998); Hamilton (1822); Kullander et al. (2009).	Hamilton (1822) reports lateral line "scarcely observable", so it's hard to discern if an abbreviated or absent lateral line conforms to description. Here, the Indian wild-caught specimen (RC0679) is referred to *D.* cf. *rerio*. Several specimens were the "leopard" variety *D. frankei* (Meinken), understood to be a selective breeding form and junior subjective synonym of *D. rerio* (Mayden et al., 2007).	EUN228 RC0067 RC0068 RC0069 RC0070 RC0071 RC0072 RC0088 RC0105 RC0394 RC0679 YGN413

Danio roseus Fang & Kottelat	As *D. albolineatus*, but: smaller; slimmer; slightly shorter barbels; posterior light and dark P/I stripes absent or v. indistinct.	Fang and Kottelat (1999, 2000).	The *D. albolineatus* complex is poorly characterised and requires systematic attention.	RC0126<br>RC0127<br>RC0128<br>RC0547<br>RC0548
Danio sp. "hikari"	Two pairs barbels (rostral extends past operculum, maxillary beyond pectoral); 7½ branched dorsal rays; lateral line incomplete; two complete lateral stripes (P and P+1) with two light interspaces, not joining in a loop behind the operculum; distinct D-stripe, A-stripe and A-1 stripe.	Smith (1931).	Similar to *D. kerri*, but likely an undescribed species.	EUN039<br>RC0262<br>RC0263<br>RC0264<br>RC0265<br>RC0266
Danio tinwini Kullander & Fang	One pair barbels (maxillary); lateral line absent; 6½ branched dorsal rays; 3 P-stripes, broken into rows of discrete spots; anal, dorsal and pelvics spotted.	Fang (1998); Kullander and Fang (2009b).		RC0062<br>RC0063<br>RC0158<br>RC0159<br>RC0160<br>YGN426<br>YGN511
Danionella dracula Britz, Conway & Rüber	Scales absent; miniature size (up to 17 mm SL); remnant larval caudal fin-folds; 13 total anal rays; 16 principal caudal rays; genital papilla not developed as a conical projection; body transparent with yellow/green lateral stripe (life).	Britz (2009); Britz et al. (2009); Roberts (1986)		YGN118
Devario cf. *acuticephala* (Hora)	Barbels absent; lateral line absent; supraorbital groove present; 10½ branched anal rays; caudal not truncate; pectorals not pointed and not reaching pelvic base; broad longitudinal stripe; no markings on fins.	Barman (1991); Hora (1921); Hora and Mukerji (1934); Talwar and Jhingran (1991).	Specimen in poor condition, and identification therefore tentative. Does not disagree with *D. acuticephala*.	RC0115
Devario cf. *aequipinnatus* (McClelland)	Two pairs barbels (rostral longer than maxillary); lateral line complete (31–36 pored scales); infraorbital process IO1 present; 10½–11½ branched dorsal rays; 12½–13½ branched anal rays; cleithral spot round and well defined; P-stripes interrupted anteriorly; P-stripe extending onto median caudal rays.	Barman (1984a); Day (1875); Fang (1997b, 2000); Jayaram (1991); McClelland (1839); Talwar and Jhingran (1991).	Identification tentative, as the concept of *D. aequipinnatus* varies considerably among authors, and is poorly characterised: following Day (1875) here.	RC0349<br>RC0350<br>RC0351<br>RC0352<br>RC0464
Devario auropurpureus (Annandale)	Barbels absent; snout sharply pointed; narrow elongate body; origin of dorsal slightly anterior to anal; lateral line complete (approx. 37 pored scales); branched dorsal rays 7½; branched anal rays 14½–16½; approx. 14 bluish (life) lateral bars; fine dark granulation on fins.	Annandale (1918); Barman (1984b).		RC0610<br>RC0689<br>RC0691<br>YGN246<br>YGN398<br>YGN485<br>YGN509
Devario cf. *browni* (Regan)	Two pairs barbels (v. small); infraorbital process IO1 present; lateral line complete (approx. 32 pored scales); branched dorsal rays 9½–10½; branched anal rays 12½–13½; predorsal scales 14–15; cleithral spot present; 3 wavy P-stripes (P-stripe continues onto caudal).	Fang (2000); Fang and Kullander (2009); Regan (1907).	Tentative identification: not entirely consistent with characters of *D. browni* presented by Fang (2000). The P+1 and P–1 stripes should meet to form a loop anteriorly: this character is not present in all material here, and the loop is positioned too far anteriorly for *D. browni* (above end of pectorals). Fin ray counts are reported to be quite varied in different populations of *D. browni* (Fang, 2000).	RC0196<br>RC0197<br>RC0198<br>RC0199<br>RC0200<br>YGN154
Devario cf. *chrysotaeniatus* (Chu)	Two pairs barbels (rostral approx. ½ eye diameter, maxillary tiny); infraorbital process IO1 present; branched dorsal rays 7½–8½; branched anal rays 12½; cleithral spot present; dorsal and anal with faint median stripe; P-stripe strong: starting above pelvics and continuing onto caudal; weak P+1 and P+2 stripes; interspace stripes break up anteriorly into dots.	Fang (2000); Fang and Kottelat (1999); Kottelat (2001).	Tentative identification: *D. chrysotaeniatus* should not have a process on infraorbital IO1. Alternative identification could be *D. laoensis* (Pellegrin & Fang).	RC0258<br>RC0259<br>RC0261
Devario cf. *devario* (Hamilton)	One pair barbels (small); lateral line complete (44–46 pored scales); infraorbital process IO1 absent; 15½–16½ branched dorsal rays; 16½–17½ branched anal rays; deep rhomboidal body shape; cleithral spot absent; three stripes on posterior of body (blue in life); network of spots and stripes in anterior of body (blue and yellow in life).	Conway et al. (2009); Hamilton (1822); Talwar and Jhingran (1991).	*Devario devario* is reported as having no barbels. This material has small but obvious barbels, so may not be conspecific with *D. devario*.	RC0510<br>RC0585<br>RC0586<br>RC0587

Devario malabaricus (Jerdon)	Two pairs barbels; lateral line complete (36–40 pored scales); infraorbital process IO1 absent; 11½ branched dorsal rays; 14½–15½ branched anal rays; snout pointed; cleithral spot present as vertical mark; 4–5 lateral stripes breaking up into spots anteriorly (blue in life).	Jayaram (1991); Jerdon (1849); Kottelat and Pethiyagoda (1990); Talwar and Jhingran (1991).		RC0406 RC0407 RC0408 RC0409 RC0410 RC0462 RC0733
Devario pathirana (Kottelat & Pethiyagoda)	Two pairs barbels; lateral line complete; infraorbital process IO1 present; 7–11 irregular parallel bars (dark blue in life); longitudinal stripe on caudal peduncle continuing onto median caudal rays; dark median stripe in dorsal.	Kottelat and Pethiyagoda (1990).		RC0529 RC0530 RC0692 RC0693
Devario sondhii (Hora & Mukerji)	Barbels absent; lateral line incomplete (8–10 pored scales); supraorbital groove present; dorsal 7½ branched rays; cleithral spot present; iridescent lateral stripe on posterior of body; sides covered with small pigmented dots; no markings on fins.	Hora and Mukerji (1934).		RC0113 RC0114 RC0165 RC0166 RC0167
Devario sp. "giraffe"	Two pairs barbels (v. small); infraorbital process IO1 present; deep, bulky body shape; lateral line complete (approx. 31–34 pored scales); branched dorsal rays 9½–11½; branched anal rays 12½–14½; predorsal scales 14–15; cleithral spot not distinct; P-stripes and interspaces broken up anteriorly into spots, rings and vertical bars.	Cottle (2010); Fang (2000); Fang and Kottelat (1999); Fang and Kullander (2009); Kottelat (2001); Regan (1907).	Presented here as an undescribed species: does not match literature, although many nominal *Devario* spp. are very poorly known. Appears very similar to *D.* sp. "giraffe" and *D.* cf. *malabaricus* as presented by Cottle (2010).	EUN042 RC0257 RC0260 RC0511 RC0634 RC0635 RC0687 RC0694 RC0695
Devario sp. "purple cypris"	Barbels absent; snout blunt, round; supraorbital groove present; infraorbital process IO1 absent; lateral line complete; approx. 9–10 lateral bars; fine dark granulation on fins (no stripes).	Annandale (1918); Barman (1984b); Fang (1997a); Fang and Kottelat (1999).	Presented here as an undescribed species: does not match literature, although many nominal *Devario* spp. are poorly known.	RC0250 RC0251 RC0252 RC0253
Devario sp. "TW04"	Barbels absent; infraorbital process IO1 absent; lateral line complete (approx. 33 pored scales); branched dorsal rays 9½; branched anal rays 10½; predorsal scales 14; cleithral spot absent; three P-stripes, with P+1 and P–1 stripes joining irregularly; two rows of metallic pink coloured scales along dorsal midline.	Cottle (2010); Fang (2000); Fang and Kottelat (1999).	Unable to confidently place to known species. Strong visual match to *D.* sp. "TW04" as presented in Cottle (2010).	YGN072
Devario sp. "undet. (1)"	Two pairs barbels (rostral longer than maxillary, and less than half eye width); lateral line complete (29–30 pored scales); infraorbital process IO1 present; 11½–12½ branched dorsal rays; 12½–13½ branched anal rays; cleithral spot present; 4–5 P-stripes, breaking up anteriorly; P-stripe wider, and extending onto median caudal rays; dusky median stripe in dorsal.	Fang (1997b, 2000); Fang and Kottelat (1999); Kottelat (2001); Myers (1924).	Literature unable to discriminate. *Devario acrostomus* (Fang and Kottelat) and *D. kakhienensis* (Anderson) are similar. Conservatively, it is presented as an undetermined (i.e. an unidentified or undescribed) species. Many nominal *Devario* spp. are poorly known. Sold as *D. strigillifer* (Myers).	RC0187 RC0188 RC0189 RC0190
Devario sp. "undet. (2)"	Two pairs barbels (rostral longer than maxillary); lateral line complete (30–32 pored scales); infraorbital process IO1 present; 9½–11½ branched dorsal rays; 10½–11½ branched anal rays; cleithral spot present; 3–4 P-stripes; P-stripe wider, and extends onto median caudal rays; bright green/yellow colouration (life).	Fang (1997b, 2000); Kottelat (2001); Myers (1924).	Possibly conspecific with *D. kakhienensis* (Anderson), but not positive enough to apply the name. Conservatively, it is presented as an undetermined (i.e. unidentified or undescribed) species. Many nominal *Devario* spp. are poorly known. Purportedly sourced from Myanmar, and sold as *D.* sp. "fluoro" or "Himalayan lemon".	RC0480 RC0481 RC0531 RC0532 RC0533
Eirmotus furvus Tan & Kottelat	Barbels absent; mouth terminal; cephalic papillae present on head (arranged in rows); lateral line incomplete; last unbranched dorsal ray serrated; 8 dark conspicuous bars, with width of bar 5 greater than 1½ scales; mark on posterior of dorsal adjacent to bar 6; last unbranched dorsal ray entirely pigmented; distinct black mark anterior to anus; back upper margin of pectoral; body and fins dusky with scattered chromatophores on fin rays.	Tan and Kottelat (2008).		YGN345

Eirmotus cf. *insignis* Tan & Kottelat	Barbels absent; mouth terminal; cephalic papillae present on head (arranged in rows); lateral line incomplete (2–6 pored scales); last unbranched dorsal ray serrated (approx. 21 serrae); 8 dark bars, with width of bar 5 approx. 1–1½ scales; row median dark spots on dorsal; mark on posterior of dorsal adjacent to bar 6; unbranched dorsal rays entirely pigmented; last unbranched anal ray pigmented in some specimens.	Tan and Kottelat (2008).	Identification tentative, as pigmentation on last unbranched dorsal and anal rays extending entire length of ray rather than proximal half/base. Diagnoses in Tan and Kottelat (2008) difficult to reconcile with these specimens.	EUN052<br>RC0667<br>RC0668<br>YGN050
Eirmotus cf. *octozona* Schultz	Barbels absent; mouth terminal; cephalic papillae present on head (arranged in rows); lateral line incomplete; last unbranched dorsal ray serrated (approx. less than 20 serrae); 8 dark bars, with width of bar 5 approx. 1 scale; row median dark spots on dorsal absent; unbranched dorsal rays entirely pigmented; unbranched anal rays unpigmented.	Tan and Kottelat (2008).	Identification tentative, as count of unbranched dorsal ray serrae fall short of the 25–31 expected in *E. octozona*. Diagnoses in Tan and Kottelat (2008) difficult to reconcile with these specimens.	YGN077<br>YGN233
Epalzeorhynchos bicolor (Smith)	Two pairs barbels (black); fimbriate rostral cap with free lateral lobe not terminating in sharp tubercle; upper lip poorly developed; lower lip not papillose; body and fins uniform dark colour; caudal orange/red (life); dorsal with white edge; dark spots behind operculum and above pectorals.	Kottelat et al. (1993); Roberts (1989); Smith (1931); Zhang and Kottelat (2006).		EUN080<br>RC0321<br>RC0322<br>YGN019
Epalzeorhynchos frenatum (Fowler)	Two pairs barbels; fimbriate rostral cap with free lateral lobe not terminating in sharp tubercle; upper lip poorly developed; lower lip not papillose; dark blotch at caudal base; no black or white margin to dorsal, pelvic and pectoral; all fins dusky orange/red (life).	Kottelat (1998, 2001); Rainboth (1996); Roberts (1989); Zhang and Kottelat (2006).		EUN081<br>RC0213<br>RC0214<br>YGN032
Epalzeorhynchos kalopterus (Bleeker)	Two pairs barbels (rostral black, maxillary pale); fimbriate rostral cap with free lateral lobe terminating in sharp tubercle; upper lip poorly developed; lower lip not papillose; well defined, broad lateral stripe (snout tip to median caudal rays).	Kottelat et al. (1993); Roberts (1989); Zhang and Kottelat (2006).		EUN079<br>RC0519<br>RC0520<br>YGN061<br>YGN127<br>YGN373<br>YGN400<br>YGN489
Esomus metallicus Ahl	Two pairs barbels (rostral extending past eye, maxillary extending past pelvic base); supraorbital groove absent; lateral line single and incomplete (extends to approx. between pelvic and anal); lateral stripe and more intense posteriorly, terminating at caudal base; no markings on fins.	Fang (2003); Hora and Mukerji (1928); Kottelat (2001); Talwar and Jhingran (1991); Tilak and Jain (1990).		RC0653<br>RC0654<br>RC0655<br>RC0656<br>RC0657<br>YGN090
Garra cambodgiensis (Tirant)	Mouth inferior; upper and lower lips continuous, with lower lip modified into sucking disc; snout tuberculated; one pair barbels (rostral); wide midlateral stripe (approx. 2 scales width); two dark bands (proximal and distal) in dorsal; caudal plain with red margins (life).	Kottelat (2001); Rainboth (1996).		RC0716<br>RC0717
Garra cf. *ceylonensis* Bleeker	Mouth inferior; ventral surface of head and body flattened; upper and lower lips continuous, with lower lip modified into sucking disc; proboscis absent; two pairs barbels; lateral line complete (32 pored scales); dark spot on gill opening; distance of anus from anal fin origin less than 4× in distance between pelvic fin origin and anal fin origin; interorbital width greater than 0.5× HL; dark spots at dorsal base absent; dark midlateral stripe with several narrow light and dark longitudinal stripes posteriorly.	Menon (1964); Talwar and Jhingran (1991)	Tentative identification as many *Garra* spp. are poorly known. Keys out as *G. ceylonensis* in Talwar and Jhingran (1991), but *G. mullya* Sykes is a plausible alternative identification, a species with a wider distribution.	YGN399
Garra flavatra Kullander & Fang	Mouth inferior; ventral surface of head and body flattened; upper and lower lips continuous, with lower lip modified into sucking disc; proboscis absent; lateral line complete (28 pored scales); 7½ branched dorsal rays; shallow rostral furrow; rostral lobe present; tubercles on rostral lobes and snout; abdomen scaled; black spot at gill opening; 3 yellow contrasting bars (life); wide, dark distal band and white tip to dorsal; subdistal band to caudal; spots on caudal.	Kullander and Fang (2004).		EUN163<br>RC0317<br>RC0318<br>YGN016<br>YGN155<br>YGN376
Garra gotyla (Gray)	Mouth inferior; ventral surface of head and body flattened; upper and lower lips continuous, with lower lip modified into sucking disc; two pairs barbels; upper lip not tuberculate; chest and ventral surface scaled; no distinct proboscis or rostral fold; lateral line complete (31–32 pored scales); 8½ branched dorsal rays; dark blotch/bar at caudal base; longitudinal stripes on posterior of body; dark posterior margin to dorsal and caudal; red/pinkish fins (life).	Menon (1964); Talwar and Jhingran (1991); Vishwanath et al. (2007).	Individuals appear juvenile, and lacking proboscis.	YGN062<br>YGN166<br>YGN219<br>YGN478<br>RC0390<br>RC0391

Garra gravelyi (Annandale)	Mouth inferior; ventral surface of head and body flattened; upper and lower lips continuous, with lower lip modified into sucking disc; unilobed indistinct square proboscis; transverse groove across upper lip; two pairs barbels (maxillary shorter than rostral); 8½ branched dorsal rays; lateral line complete (32 pored scales); 8 predorsal scales; dark spot on gill opening; dark spots at dorsal base; dark midlateral stripe.	Kottelat (2000); Menon (1964).	Unable to count diagnostic circumpeduncular scales due to tissue excision from this area: estimated from photograph to be approx. 12.	RC0272<br>RC0273<br>YGN046
Garra rufa (Heckel)	Mouth inferior; ventral surface of head and body flattened; upper and lower lips continuous, with lower lip modified into sucking disc; lateral line complete (35 pored scales); proboscis absent; 8½ branched dorsal rays; 17 branched caudal rays; 4–5 dark spots at base of dorsal; black spot at upper opening of operculum; dark blotch at caudal base; lower lobe of caudal dark; darkly mottled flanks.	Coad (2010); Menon (1964).		RC0526<br>RC0527<br>YGN105<br>YGN159<br>YGN199
Garra sp. "undet. (1)"	Mouth inferior; ventral surface of head and body flattened; upper and lower lips continuous, with lower lip modified into sucking disc; proboscis absent; two pairs barbels; snout rounded; lateral line complete (approx. 33 pored scales; 8½ branched dorsal rays; no spots at dorsal base; dark bar at base of caudal; fins with no distinct markings; no longitudinal stripes posteriorly; no spot behind gill opening; fins with no distinct markings.	Menon (1964); Talwar and Jhingran (1991); Vishwanath et al. (2007).	Unable to confidently place to known species. *G. annandalei* Hora and *G. manipurensis* Vishwanath & Sarojnalini appear close.	RC0386<br>RC0387
Gyrinocheilus aymonieri (Tirant)	Spiracle above operculum; dorsal with 9½ branched rays; caudal spotted; dark spot posterior to spiracle.	Roberts and Kottelat (1993).	*Gyrinocheilus* is a gyrinocheilid.	EUN164<br>RC0395<br>RC0396<br>YGN018<br>YGN033<br>YGN230
Hampala macrolepidota Kuhl & van Hasselt	One pair barbels; mouth large, extending past anterior margin of eye; last unbranched dorsal ray finely serrated; lateral line complete (25–27 pored scales); narrow black bar between dorsal and anal origin; black bar on caudal peduncle; caudal red (life) with black submarginal stripes.	Doi and Taki (1994); Inger and Chin (1962); Kottelat (1998, 2001); Ryan and Esa (2006).	Discrepancies in lateral line scale counts and presence of black markings on posterior of body make identification as *H. macrolepidota* tentative. However, inconsistency between authors suggest the name be maintained here as most likely identification. Specimens were immature.	RC0367<br>RC0368
Hypsibarbus wetmorei (Smith)	Lateral line complete; 4½ scales between lateral line and dorsal origin; 2 rows of scales between anus and anal origin; last unbranched dorsal ray serrated; distance between distal dorsal serrae greater than width of their base; 8 branched pelvic rays; shallow groove in lower lip between jaw; dark scale bases, reticulated pattern; pectorals, pelvics and anal yellow/orange colour (life).	Kottelat (2001); Rainboth (1996).	Unable to count circumpeduncular scales, so cannot entirely rule out *H. malcolmi* (Smith).	RC0180<br>RC0181<br>YGN430
Labeo cf. *boga* (Hamilton)	One pair minute maxillary barbels; upper lip covered by rostral cap; lateral line complete (38 pored scales); 4½ scales between lateral line and pelvic base; 9½ branched dorsal rays; 5½ branched anal rays; dark spot above pectoral; dark bar on caudal peduncle.	Hamilton (1822); Talwar and Jhingran (1991).	Identification tentative, as literature cannot rule out alternative such as *L. ariza* (Hamilton), *L. bata* (Hamilton) and *L. kawrus* (Sykes). Most likely *L. boga*, however.	RC0671<br>RC0672
Labeo chrysophekadion (Bleeker)	Two pairs barbels; lips fimbriated; upper lip covered by rostral cap with broad lateral folds; dorsal large, with straight margin and 18½ branched rays; black body and fin colour.	Kottelat (2001).		RC0369<br>RC0370
Labeo cyclorhynchus Boulenger	Two pairs barbels (maxillary large and visible); lips plicate; snout large and rounded; upper lip covered by broad rostral cap; dorsal deeply concave with 12½ branched rays; variegated body colour pattern.	Tshibwabwa et al. (2006); Tshibwabwa and Teugels (1995).		RC0506<br>RC0507
Labiobarbus leptocheilus (Valenciennes)	Two pairs barbels (maxillary extending to not beyond centre of eye, rostral short); lips fimbriated; lateral line complete (36 pored scales); long dorsal fin (24½ branched rays); 5½ branched anal rays; approx. 10 rows spots forming longitudinal stripes.	Kottelat (2001); Roberts (1994).		RC0376
Labiobarbus ocellatus (Heckel)	Two pairs barbels; lips plicate; scales small (61 pored lateral line scales); long dorsal fin (28½ branched rays); no lateral stripes; ocellated humeral spot; ocellated spot on caudal peduncle and caudal base; fins without markings.	Kottelat et al. (1993); Roberts (1994).		RC0274<br>RC0275
Leptobarbus rubripinna (Fowler)	Two pairs barbels (maxillary barbel not reaching past centre of eye); lateral line complete, terminating on ventral half of caudal peduncle; 4½ scales between lateral line and dorsal origin; 7½ branched dorsal rays; no back blotch posterior to operculum; black midlateral stripe approx. ½–1 scale width; caudal lobes without black submarginal stripes; pelvic, anal, caudal red/orange (life).	Kottelat (2001); Kottelat et al. (1993); Rainboth (1996); Roberts (1989); Tan and Kottelat (2009).		RC0296<br>RC0460
Leuciscus idus (Linnaeus)	Barbels absent; mouth terminal; lateral line complete (53–56 pored scales); 8½–9½ branched dorsal rays; 11½ branched anal rays; posterior margin of anal concave.	Kottelat and Freyhof (2007).	Ornamental blue variety.	RC0570<br>RC0571
Luciosoma setigerum (Valenciennes)	Two pairs barbels (well developed); mouth large; snout strongly pointed; 7½ branched dorsal rays; 6½ branched anal rays; pelvic filaments extend to anal origin; semicircle of tubercles between nostrils absent; scattered tubercles on lower jaw and snout; dorsal positioned in posterior half of body; dark spots on caudal absent; midlateral stripe of indistinct spots, continuing onto caudal as submarginal stripe of upper lobe; median caudal rays not pigmented.	Kottelat (2001); Kottelat et al. (1993); Rainboth (1996); Roberts (1989).		RC0294<br>RC0295<br>YGN026<br>YGN488

Microdevario kubotai (Kottelat & Witte)	Barbels absent; lateral line absent; predorsal scales 10; narrow infraorbital 4; 7½ branched dorsal rays; 9½–10½ branched anal rays; concave distal margins of anal and dorsal; wide midlateral stripe, diffuse anteriorly; cleithral spot absent; no stripes on fins; black anal papilla absent; thin axial streak from above anus to caudal base.	Fang et al. (2009); Jiang et al. (2008); Kottelat and Witte (1999).		RC0234<br>RC0235<br>RC0492<br>RC0601<br>RC0602<br>YGN510
Microdevario nana (Kottelat & Witte)	As *M. kubotai*, but: distinct dark spot on tip of dorsal; diffuse spot on tip of anal; 10½–11½ branched anal rays; thin midlateral stripe, diffuse anteriorly; unpaired fins yellowish (life).	Fang et al. (2009); Jiang et al. (2008); Kottelat and Witte (1999).		EUN161<br>RC0618<br>RC0619<br>RC0620<br>RC0621<br>RC0622
Microrasbora rubescens Annandale	Barbels absent; supraorbital groove present; wide infraorbital 4; lateral line absent; predorsal scales 13; 7½–8½ branched dorsal rays; 10½–11½ branched anal rays; cleithral spot absent; no stripes on fins; black anal papilla; bright orange/red colouration with greenish lateral stripe (life).	Annandale (1918); Cottle (2010); Fang (2003); Fang et al. (2009); Jiang et al. (2008); Kottelat and Witte (1999).	These are a smaller, narrower, more colourful fish (2.8 cm TL), and perhaps better fit the description of *M. rubescens* (Annandale, 1918) than the *M.* cf. *rubescens* specimens. Found as possible bycatch with another lake Inle species, *Danio erythromicron*.	EUN162<br>RC0662
Microrasbora cf. *rubescens* Annandale	As *Microrasbora rubescens*, but: larger (4.3 cm TL), deeper bodied and bulkier; duller pinkish/orange hue (life).	Annandale (1918); Cottle (2010); Fang (2003); Fang et al. (2009); Jiang et al. (2008); Kottelat and Witte (1999).	These are larger fish than described by Annandale (1918). They are also less colourful. It is not exactly clear which of the *M. rubescens* specimens here are conspecific with the types, but these a poorer fit than the other specimens (RC0662, EUN162), and so are regarded for now as *M.* cf. *rubescens*. Additionally, Fang (2003) reports the supraorbital groove absent in her *M. rubescens* material. Very similar in appearance to *Devario* sp. "TW04" as presented by Cottle (2010).	RC0681<br>RC0682<br>RC0683<br>RC0684<br>RC0685
Mystacoleucus argenteus (Day)	Two pairs barbels; lateral line complete; procumbent predorsal spine; body deep and laterally compressed; eyes large; 8½ branched dorsal rays; last unbranched dorsal ray serrated; 6½ branched anal rays; dorsal origin anterior to pelvic origin; anal with concave distal margin; dorsal with black distal margin, becoming fainter posteriorly; strong black margin to caudal absent; dark scale base crescents absent.	Kottelat (2001); Talwar and Jhingran (1991).		EUN049
Myxocyprinus asiaticus (Bleeker)	Barbels absent; mouth small and inferior; lips papillated; ventral surface flat; high body, strongly laterally compressed; dorsal origin just posterior to pectoral base; dorsal, sail-like, terminating at caudal peduncle; variegated colouration with 4 dark bars.	Gao et al. (2008).	*Myxocyprinus* is a catostomid.	RC0203<br>RC0204
Neolissochilus cf. *stracheyi* (Day)	Two pairs barbels; lateral line complete (24+2 pored scales); last unbranched dorsal spine not serrated; 9½ branched dorsal rays; post labial groove interrupted (no median fleshy lobe on lower lip); tubercles on sides of snout and below eye; 3½ rows scales between dorsal origin and lateral line; dark midlateral stripe; back bronze and belly silver (life).	Chen et al. (1999); Day (1875); Kottelat (2001); Vidthayanon and Kottelat (2003).	Systematics of *Neolissochilus* is confused. Both *N. baoshanensis* (Chen & Yang) and *N. wynaadensis* (Day) are possible identifications, but tentatively, *N.* cf. *stracheyi* appears the most likely fit.	RC0365
Opsarius bakeri (Day)	One pair barbels (minute); lateral line complete; 10½–11½ branched dorsal rays; 13½ branched anal rays; single row 10–12 midlateral short bars/spots, becoming more elongated anteriorly; anal, dorsal and pelvics with black distal and white proximal stripes; caudal with white margins to lobes, and upper lobe with submarginal black blotch anteriorly.	Day (1865); Remi Devi et al. (2005); Talwar and Jhingran (1991).	Generic nomenclature follows Tang et al. (2010).	RC0377<br>RC0378
Oreichthys cosuatis (Hamilton)	Barbels absent; snout pointed; scales between pelvic origin and dorsal midline: ½, 6, ½; cephalic papillae present on head (arranged in rows); lateral line incomplete (4–5 pored scales); last unbranched dorsal ray not serrated; 8½ branched dorsal rays; 5½ branched anal rays; scales with dark bases: reticulate pattern; no spot on caudal peduncle; anal with indistinct median stripe/blotch; black subdistal margin on dorsal.	Schäfer (2009).	Schäfer (2009) reports 2–3 pored lateral line scales.	RC0470<br>RC0471
Oreichthys crenuchoides Schäfer	Barbels absent; snout blunt; scales between pelvic origin and dorsal midline: ½, 7, ½; cephalic papillae present on head (arranged in rows); lateral line incomplete; last unbranched dorsal ray not serrated; 8½ branched dorsal rays; 5½ branched anal rays; scales with dark bases: reticulate pattern; no spot on anal; spot on caudal base greater than ⅓ of peduncle depth; distal-anterior blotch on dorsal in females.	Schäfer (2009).		RC0050<br>RC0051

Oreichthys parvus Smith	Barbels absent; snout pointed; scales between pelvic origin and dorsal midline: ½, 6, ½; cephalic papillae present on head (arranged in rows); lateral line incomplete (6 pored scales); last unbranched dorsal ray not serrated; 8½ branched dorsal rays; 5½ branched anal rays; scales with dark bases: reticulate pattern; spot on caudal base less than ⅓ of peduncle depth; anal with spot; dark marking on tip of dorsal.	Schäfer (2009).		EUN207
Oreichthys sp. "red fin"	Barbels absent; snout blunt; scales between pelvic origin and dorsal midline: ½, 6, ½; cephalic papillae present on head (arranged in rows); lateral line incomplete (5–6 pored scales); last unbranched dorsal ray not serrated; 8½ branched dorsal rays; 5½ branched anal rays; scales with dark bases: reticulate pattern; blotch covering almost entire caudal peduncle; anal with spot; anterior subdistal blotch on dorsal continuing as median stripe (females), with no spot on dorsal in male; red colouration on body, caudal, dorsal and pelvics, anal in males (life).	Schäfer (2009).	Differs from *O. parvus* in snout shape and size of blotch on caudal base. Likely an undescribed species.	RC0638<br>RC0639
Osteochilus bleekeri Kottelat	Two pairs barbels; lips plicate; dorsal strongly concave anteriorly (11½ branched rays); last unbranched dorsal ray not serrated; 5½ branched anal rays; black blotch on proximal-anterior of dorsal; 6–7 rows lateral spots.	Kottelat (2008a); Kottelat et al. (1993); Roberts (1994).		RC0276<br>RC0659
Osteochilus microcephalus (Valenciennes)	Two pairs barbels; lips fimbriated and folded; mouth subinferior; tubercle at end of snout; 22 gill rakers; dorsal with 11½ branched rays; last unbranched dorsal ray not serrated; 5½ branched anal rays; wide midlateral stripe from operculum to caudal base; two rows of spots on dorsal.	Kottelat (2001, 2008a); Kottelat and Tan (2009); Kottelat et al. (1993); Roberts (1989).	More gill rakers (27–35) are reported by Kottelat (2008a), but fishes here are juveniles.	RC0217<br>RC0218
Osteochilus vittatus (Valenciennes)	Two pairs barbels; lips fimbriated and folded; mouth subinferior; snout tubercles absent; 5½ scale rows between dorsal origin and lateral line; last unbranched dorsal ray not serrated; scale rows with dark spots forming faint stripes; midlateral stripe absent; medium-sized blotch on caudal peduncle; fins red colour (life).	Kottelat (2001); Kottelat et al. (1993); Tan and Kottelat (2009)	Identification tentative as unable to count circumferential scales rows, so cannot effectively distinguish between *O. vittatus* and *O. kappenii* Bleeker. Specimens were wild-caught in Singapore, so based on distribution, *O. vittatus* is a more likely occurrence.	EUN038<br>YGN045
Paedocypris cf. *carbunculus* Britz & Kottelat	Scales absent; miniature size (up to 10 mm SL); modified pelvic fin in males forming keratinised "flange and hook" on anterior ray; pre-anal larval fin fold in females; single irregular row of mid-dorsal chromatophores; head blotch v-shaped; head-kidney pigment present; chest spots present; well developed chest blotch; opercular and branchiostegal rows of pigment; lips not heavily pigmented; red colouration (life).	Britz and Kottelat (2008); Kottelat et al. (2006).	*Paedocypris carbunculus* should have three rows of mid-dorsal chromatophores, and does not have a v-shaped head blotch (Britz and Kottelat, 2008). Likely an undescribed species, but conservatively regarded here as *P.* cf. *carbunculus*.	RC0222<br>RC0223
Paedocypris cf. *micromegethes* Kottelat, Britz, Tan, & Witte	Scales absent; miniature size (up to 10 mm SL); modified pelvic fin in males forming keratinised "flange and hook" on anterior ray; single row of mid-dorsal chromatophores; head-kidney pigment absent; overall, lightly pigmented; chest blotch present (distinct); red colour (life).	Britz and Kottelat (2008); Kottelat et al. (2006).	*Paedocypris micromegethes* should have a poorly developed or absent chest blotch, so these specimens are best referred as *P.* cf. *micromegethes*. Both specimens have different head blotch patterns, however, and are not regarded as conspecific with one another.	YGN554<br>EUN045
Pectenocypris korthausae Kottelat	Barbels absent; symphyseal knob present; pointed snout; elongate body shape; v. large number comb-like gill rakers (not counted); 7½ branched dorsal rays; 5½ branched anal rays; last unbranched dorsal ray not serrated; dorsal origin above pelvic; lateral line incomplete (8 pored scales); round black spot on caudal base occupying 50% of peduncle; axial streak from operculum to caudal peduncle.	Kottelat (1982); Tan and Kottelat (2009).		RC0590
Poropuntius normani Smith	Two pairs barbels; mouth inferior; lateral line complete (28 +2–3 pored scales); lateral line with accessory ventral pore; last unbranched dorsal ray serrated; well defined dark stripe along margins of caudal lobes; yellow caudal (life).	Kottelat (2000, 2001).		RC0545<br>RC0546
Puntioplites proctozystron (Bleeker)	Barbels absent; lateral line complete; last unbranched anal ray thick and serrated posteriorly; last unbranched dorsal ray short, not reaching caudal; body plain with no markings; fins without orange colour.	Kottelat (2001); Kottelat et al. (1993); Taki and Katsuyama (1979).		RC0176<br>RC0177
Puntius arulius (Jerdon)	One pair maxillary barbels; mouth subterminal; lateral line complete; last unbranched dorsal ray smooth; dark band across caudal lobes absent; three large blotches on body (>2 scales): large blotch mid body above pelvic origin, dark blotch above anal, dark blotch on caudal base; dorsal filaments absent in males.	Devi et al. (2010); Knight et al. (2011); Pethiyagoda and Kottelat (2005).		RC0555<br>RC0556<br>RC0557<br>RC0558<br>RC0559
Puntius assimilis (Jerdon)	Lateral line complete; smooth last unbranched dorsal ray; one pair maxillary barbels (long); mouth inferior; dark band across caudal lobes; dark posterior lateral blotch; no markings on body anterior to anal origin.	Devi et al. (2010); Pethiyagoda and Kottelat (2005).	Some specimens small, but salient features discernible. There is diversity in the species, with three populations tentatively treated as conspecific, plus one synonym (*P. lepidus* Day).	RC0132<br>RC0133<br>RC0134<br>RC0490<br>RC0491

Puntius aff. *banksi*	Two pairs long barbels; lateral line complete; last unbranched dorsal ray serrated; wedge-shaped marking beneath dorsal covering 3–4 scales; spot above anterior of anal; blotch on caudal peduncle.	Herre (1940); Kottelat and Lim (1995); Ng and Tan (1999); Rachmatika (2004).	Type material of *P. banksi* comprises two batches, viz. Singapore and Sarawak; Sarawak material (lectotype) comprises a species with elongate black bar at base of dorsal 1–2 scales in width, so likely not conspecific with Singapore material which matches these fish.	RC0303<br>RC0393
Puntius chalakkudiensis Menon, Rema Devi & Thobias	One pair maxillary barbels; mouth inferior; lateral line complete (28 pored scales); smooth last unbranched dorsal ray; pronounced snout; black midlateral stripe with scarlet stripe above anteriorly; caudal with oblique dark distal band; dark median spot anteriorly on dorsal.	Day (1865); Menon et al. (1999); Prasad et al. (2008); Talwar and Jhingran (1991).		RC0537<br>RC0538<br>RC0539<br>RC0540<br>RC0541
Puntius chola (Hamilton)	One pair barbels (maxillary); mouth subterminal; 8½ branched dorsal rays; spot on caudal peduncle; proximal-anterior spot on dorsal branched rays 1–4; median-proximal row of dots above spot on dorsal.	Hamilton (1822); Silva et al. (2008); Talwar and Jhingran (1991).	Individual lacks iridescent pigments.	RC0730
Puntius conchonius (Hamilton)	Barbels absent; lateral line incomplete (8–13 pored scales); 8½ branched dorsal rays; deep body; dark blotch on caudal peduncle (no anterior blotches); dorsal with thick distal band.	Hamilton (1822); Talwar and Jhingran (1991); Vishwanath et al. (2007).		RC0001<br>RC0002<br>RC0084<br>RC0156<br>RC0371<br>RC0372<br>RC0373
Puntius denisonii (Day)	One pair barbels (maxillary); lateral line complete (28 pored scales); smooth last unbranched dorsal ray; mouth inferior; no pronounced snout; black midlateral stripe with scarlet stripe above anteriorly; caudal with oblique dark distal band.	Day (1865); Menon et al. (1999); Prasad et al. (2008); Talwar and Jhingran (1991).		RC0020<br>RC0106<br>RC0119<br>RC0150<br>RC0151<br>RC0712<br>YGN015<br>YGN114
Puntius dunckeri (Ahl)	Two pairs long barbels; lateral line complete; 8½ branched dorsal rays; last unbranched dorsal ray not serrated; colour pattern: see comments.	Ahl (1929); Kottelat et al. (1993).	Kottelat et al. (1993) and Ahl (1929) report *P. everetti* (Boulenger) with five round black spots, two above lateral line and two below, with a fifth spot on the caudal peduncle, and a bar posterior to the operculum. Examination of the type series [BMNH 1893.3.6.213–218(6)] confirms this. Specimens examined here do not appear to be conspecific with *P. everetti*, and although the description of *P. dunckeri* Ahl (1929) reveals little information and no types are known, the fish illustrated superficially matches these presented there, with strikingly larger blotches, and the midlateral bar above pelvics elongated to form a distinct bar.	RC0017<br>RC0018<br>RC0145<br>RC0146<br>RC0147

Puntius erythromycter Kullander	Barbels absent; lateral line incomplete; lateral scale row curved; last unbranched dorsal ray serrated; 8½ branched dorsal rays; humeral marking absent; dark band around caudal peduncle; snout red (life).	Kullander (2008).		RC0603 RC0675 RC0676 RC0677 RC0678
Puntius fasciatus (Jerdon)	Two pairs barbels (maxillary longer than eye diam.); last unbranched dorsal ray not serrated; three scale rows between mid-dorsal row and lateral line; lateral line complete; four wide, irregular dark bars viz. oblique band between eyes, bar above pelvic, bar above anal, bar on caudal base.	Jayaram (1990); Jerdon (1849); Pethiyagoda and Kottelat (2005); Talwar and Jhingran (1991).	Possible diversity within the species, as four other names available in synonymy of *P. fasciatus*. Have chosen oldest available name in absence of modern treatment.	RC0021 RC0022 RC0101 RC0102 RC0168 RC0169 RC0170 RC0353 RC0354 YGN267 YGN395
Puntius filamentosus (Valenciennes)	One pair maxillary barbels (short); lateral line complete; last unbranched dorsal ray not serrated; mouth subterminal; dark band across caudal lobes; dark posterior lateral blotch; no markings on body anterior to anal origin.	Pethiyagoda and Kottelat (2005). Devi et al. (2010).		RC0007 RC0008 RC0116 RC0117 RC0118 RC0293 RC0299 RC0688
Puntius foerschi (Kottelat)	Two pairs barbels; lateral line complete (24 pored scales); 5½ branched anal rays; six dark bars; up to four spots between second, third and fourth bars.	Kottelat (1982); Kottelat et al. (1993).		RC0098 RC0099 RC0100 RC0665 RC0666
Puntius gelius (Hamilton)	Barbels absent; lateral line incomplete (up to 5 pored scales); last unbranched dorsal ray strongly serrated; 8½ branched dorsal rays; black band around caudal peduncle; black anterior spot on anal (not extending onto body); distinct black spots on pelvics; black spot on anterior base of dorsal; last unbranched dorsal ray not pigmented posterior to spot.	Bordoloi and Baishya (2006); Hamilton (1822); McClelland (1839); Vishwanath and Laisram (2004).	RC0135–RC0137 appear a larger fish with different form, but do not deviate significantly from the description.	RC0038 RC0039 RC0135 RC0136 RC0137 RC0604 RC0605
Puntius aff. *gelius*	Barbels absent; lateral line incomplete (up to 4 scales); last unbranched dorsal ray strongly serrated; 8½ branched dorsal rays; black band around caudal peduncle; black anterior spot on anal (extending onto body); distinct black spots on pelvics absent; black spot on anterior base of dorsal; last unbranched dorsal ray pigmented posterior to spot.	Bordoloi and Baishya (2006); Hamilton (1822); McClelland (1839); Vishwanath and Laisram (2004).	Differs from description of *P. gelius* in lacking spots on pelvics (RC0741 has v. faint marking). Also differs from my *P. gelius* in the anal fin spot extending well on to body and the pigmentation of last unbranched dorsal extending to tip (vs. not extending, and no dark pigmentation to tip). Appears as a smaller, more translucent fish. The description of *P. canius* (Hamilton) does not mention the pelvic spots, but Hamilton's illustrations published by McClelland (1839) show spots. *Puntius canius* is described as a smaller fish with a reddish hue; my material does not show a red colour, but this may be a seasonal, breeding effect. Bordoloi and Baishya (2006) report this colouration from specimens of "*P. ornatus*" Vishwanath & Laisram from Assam, and the specimens they picture appear similar, but are not *P. ornatus* as described (only markings being a band around caudal peduncle). I am reluctant to call my specimens *P. canius* or *P. ornatus*, and await further study.	RC0468 RC0469 RC0600 RC0740 RC0741

Puntius hexazona (Weber & de Beaufort)	Two pairs barbels; lateral line complete (but see comments); 5½ scales between dorsal and lateral line; six dark bars; dark spot below posterior base of dorsal absent.	Alfred (1963); Kottelat et al. (1993).	Specimens RC0361 and RC0362 appear to have incomplete lateral lines. They are referred to as *P.* cf. *hexazona*.	RC0046 RC0047 RC0048 RC0361 RC0362
Puntius jerdoni (Day)	Two pairs barbels (maxillary = eye diameter, rostral shorter); last unbranched dorsal ray not serrated; lateral line complete; 9½ branched dorsal rays; 6½ branched anal rays; 12 predorsal scales; colour silvery (life); fins orange (life) and tipped with black.	Day (1870, 1875); Talwar and Jhingran (1991).	Perhaps better referred to *Hypselobarbus*, but will follow Talwar and Jhingran (1991) in the absence of a modern treatment.	RC0611 RC0612
Puntius johorensis (Duncker)	Two pairs barbels; 4-5 dark stripes (wide, approx. 1 scale); stripes +1 and -1 on scale rows +2 and -2; no distinct axial streak below dorsal fin base.	Kottelat (1996).	Assigned as *P. johorensis*, but indistinct axial streak present on RC0641; number of stripes mostly lower than that reported by Kottelat (1996), but fits *P. johorensis* better than alternative species.	RC0379 RC0380 RC0381 RC0382 RC0383 RC0641
Puntius lateristriga (Valenciennes)	Two pairs barbels; deep body; lateral line complete; last unbranched dorsal ray serrated; two wide (2–4 scales) dark bars: first above pectoral, second wider, between dorsal and pelvics; dark midlateral stripe (1–2 scales) commencing anterior to anal, continuing onto caudal; spot above anterior of anal; RC0515 and RC0516 with more indistinct patterning comprising series of dark scale bases rather than solid lines, and midlateral stripe not extending into caudal.	Talwar and Jhingran (1991).	Six forms from the Malay Peninsula were recognised by Tweedie (1961): RC0302, RC0019 and RC0298 conform to the Johore form, while RC0515 and RC0516 conform to Perlis and Kedah form; these forms are not regarded as as conspecific in analysis, but the name *Barbus zelleri* Ahl may apply to Malay fishes.	RC0019 RC0298 RC0302 RC0515 RC0516
Puntius lineatus (Duncker)	Barbels absent; 5½ scale rows between dorsal origin and lateral line; mouth subinferior; fleshy lower lip forming continuous postlabial groove; longitudinal dark stripes.	Kottelat (1996).		EUN047
Puntius manipurensis Arunkumar & Tombi Singh	Barbels absent; lateral line incomplete (4 pored scales); 8½ branched dorsal rays; last unbranched dorsal ray serrated; small (one scale) humeral spot (not bar); small (one scale) caudal peduncle spot; 2–3 faint rows of spots in dorsal; spots absent from pelvic and anal; pigmented scale base; red colouration (life).	Arunkumar and Tombi Singh (2003); Kullander and Britz (2008); Linthoingambi and Vishwanath (2007); Menon et al. (2000).		RC0646 RC0647 RC0648 RC0649
Puntius nigrofasciatus (Günther)	Barbels absent; mouth subterminal; lateral line complete; last unbranched dorsal ray serrated; three complete dark bars above pectoral, pelvic and anal fins; oblique bar between eyes; scales with dark pigment at base.	Günther (1868); Kottelat and Pethiyagoda (1991); Pethiyagoda (1991); Talwar and Jhingran (1991).		RC0094 RC0095 RC0096 RC0149 RC0710
Puntius oligolepis (Bleeker)	One pair barbels; lateral line incomplete (6–7 pored scales); last unbranched dorsal ray not serrated; parallel rows of papillae on head; no bars or stripes; black distal margin to dorsal and anal; dark crescents along scale rows.	Kottelat et al. (1993); Tan and Kottelat (2008).		RC0014 RC0015 RC0016 RC0104 RC0311
Puntius orphoides (Valenciennes)	Two pairs barbels; last unbranched dorsal ray serrated; lateral line complete (29–31 pored scales); blotch on caudal peduncle; spot below dorsal origin; dark bar immediately anterior to operculum; caudal red with dark marginal stripes; dots along scale rows.	Kottelat (2001); Rainboth (1996).		RC0182 RC0183 RC0184 RC0185 RC0186 YGN004
Puntius padamya Kullander & Britz	One pair barbels (maxillary, small); lateral line incomplete (5–8 scales); last unbranched dorsal ray serrated; 2–3 rows dark spots on dorsal, pelvic and anal (males); vertical humeral blotch covering 3 scales; dark blotch on caudal peduncle; red colouration; base of scales heavily pigmented.	Kullander and Britz (2008).		RC0043 RC0044 RC0045 RC0152 RC0153 RC0711 YGN041 YGN056 YGN196 YGN404

Puntius pentazona (Boulenger)	Two pairs barbels; lateral line complete; 5½ scales between dorsal and lateral line; six dark bars; dark spot below posterior base of dorsal.	Alfred (1963); Kottelat et al. (1993).		RC0013<br>RC0304<br>RC0305<br>RC0306
Puntius rhomboocellatus Koumans	Two pairs barbels; lateral line complete; 5½ branched anal rays; 4½ scales between dorsal origin and lateral line; six irregular black bars with "ocellate rhombi" widening midlaterally; no spots between bars.	Alfred (1963); Kottelat (1982); Kottelat et al. (1993); Roberts (1989).		EUN232<br>RC0023<br>RC0024<br>RC0025<br>RC0154<br>RC0155<br>YGN076<br>YGN129
Puntius sahyadriensis Silas	Barbels absent; mouth subterminal; dorsal profile strongly convex; last unbranched dorsal ray not serrated, and also dark; pelvics black with white distal margins; scales with dark margin; up to seven irregular spots or vertical marks on sides.	Silas (1953).		RC0338<br>RC0339<br>RC0340<br>RC0341<br>RC0342
Puntius cf. *sarana* (Hamilton)	Two pairs barbels; lateral line complete (31+2 scales); last unbranched dorsal ray serrated; deep body; diffuse dark round blotch on caudal peduncle; rows of spots forming indistinct lateral stripes running along base of scales.	Hamilton (1822); Kottelat and Pethiyagoda (1991); Pethiyagoda (1991).	Much uncertainty this in identification, with 22 available names in the synonymy of *P. sarana*. Hamilton (1822) states two minute barbels, so maybe not this fish; here I follow Pethiyagoda (1991) and use the oldest available name pending a critical review.	RC0074
Puntius semifasciolatus (Günther)	One pair barbels, small; last unbranched dorsal ray serrated and shorter than adjacent branched ray; lateral line complete; series (up to seven) of irregular lateral marks (spots or bars), with last bar forming spot on caudal base.	Chang et al. (2006); Günther (1868); Kottelat (2001).		RC0040<br>RC0041<br>RC0042<br>RC0093<br>RC0142<br>RC0673<br>RC0674
Puntius shalynius Yazdani & Talukdar	Barbels absent; lateral line incomplete (up to 11 pored scales); dark axial streak; last unbranched dorsal ray strongly serrated; 7½ branched dorsal rays; prominent first dark spot on peduncle above posterior of anal; indistinct second spot on caudal base; base of scales dark.	Yazdani and Talukdar (1975).	Yazdani and Talukdar (1975) reports orange/black fins, perhaps this material is immature?	RC0485<br>RC0486<br>RC0487<br>RC0488<br>RC0489
Puntius cf. *sophore* (Hamilton)	Barbels absent; mouth terminal; lateral line complete; last unbranched dorsal ray smooth; 8½ branched dorsal rays; dark proximal spot on branched dorsal rays 3, 4 and 5; dark spot on caudal peduncle and base; golden blotch on operculum; pelvic and anal yellow (life).	Hamilton (1822); Silva et al. (2008); Talwar and Jhingran (1991).	Much uncertainty in identification, with five available names in synonymy of *P. sophore*. Hamilton (1822) states four minute barbels, so probably not this fish. *Puntius stigma* (Valenciennes) may apply here, but I conservatively use the diagnosis of Talwar and Jhingran (1991), citing the oldest available name pending a critical review.	RC0658<br>RC0729
Puntius sp. "hybrid"	See comments.		Purported to be a hybrid of *P. denisonii* and *P. everetti*. Does not convincingly match any known *Puntius* species. The presence of a weak red stripe above the black midlateral stripe suggests *P. denisonii* may indeed be a parent.	RC0171<br>RC0172<br>RC0173<br>RC0174<br>RC0175
Puntius stoliczkanus (Day)	Barbels absent; lateral line complete; 8½ branched dorsal rays; last unbranched dorsal ray serrated (11-16 serrae); black vertical blotch on scales 3–4 above pectoral; black blotch on caudal peduncle; 2 black rows of spots on dorsal.	Hamilton (1822); Kottelat (2001); Linthoingambi and Vishwanath (2007).		RC0473<br>RC0474<br>RC0512<br>RC0576<br>RC0577<br>RC0718

Puntius tambraparniei Silas	One pair barbels; mouth terminal; lateral line complete; last unbranched dorsal ray not serrated; dark band across caudal lobes absent; four large blotches on body: two dark narrow bars under dorsal; dark blotch above anal, dark bar on caudal base; dorsal filaments present in males.	Devi et al. (2010); Knight et al. (2011); Pethiyagoda and Kottelat (2005).	Some specimens small, but salient features discernible.	RC0010<br>RC0011<br>RC0012<br>RC0097<br>RC0528<br>RC0732
Puntius tetrazona (Bleeker)	One pair barbels; last unbranched dorsal ray serrated; lateral line incomplete; four vertical dark bars; dark proximal band on dorsal not extending onto body.	Alfred (1963); Kottelat et al. (1993).	Specimens here have an incomplete lateral line, but with 10–13 pored scales. Kottelat et al. (1993) reports 8–9 pored scales for *P. tetrazona*, and illustrates a fish with black pelvics (as does BMNH syntype 1867.11.28.178), but there is no mention on this in the literature. Identified as *P. tetrazona* (Bleeker) over *P. anchisporus* (Vaillant). Additional material (RC0742–RC0743) has 6–7 pored scales and 12 circumpeduncular scales, also conforming to *P. tetrazona*. Photos of wild (live) *P. anchisporus* with a clearly complete lateral line are nearly identical looking to the aquarium tiger barb. Photos of wild putative *P. tetrazona* with black pelvics are a quite different looking fish, although there has been a long history of selective breeding this fish. Retained for time being as *P. tetrazona*.	EUN103<br>EUN233<br>RC0004<br>RC0005<br>RC0006<br>RC0083<br>RC0140
Puntius tiantian Kullander & Fang	One pair barbels (maxillary, rudimentary); mouth subterminal; lateral line complete; 8½ branched dorsal rays; last unbranched dorsal ray thin and weakly serrated; large dark humeral bar; large dark blotch on caudal peduncle forming indistinct band.	Kullander and Fang (2005).		RC0501<br>RC0502<br>RC0503<br>RC0504<br>RC0505
Puntius ticto (Hamilton)	Barbels absent; lateral line incomplete (up to 11 pored scales); 24 scales in lateral series; 8½ branched dorsal rays; last unbranched dorsal ray serrated (13–15 serrae); dark spot on 3rd–4th lateral line scale; dark midlateral blotch above posterior of anal (on 17th–19th lateral scale); 1–2 rows of irregular spots on dorsal.	Hamilton (1822); Linthoingambi and Vishwanath (2007); Menon et al. (2000).	Linthoingambi and Vishwanath (2007) reports 15–17 serrae on last unbranched dorsal ray. *Puntius ticto* appears to vary geographically, and may comprise a complex of species.	RC0623<br>RC0624<br>RC0625
Puntius titteya Deraniyagala	One pair barbels; incomplete lateral line (3–5 pored scales); last unbranched dorsal ray weakly serrated; dark midlateral stripe from lip extending into caudal; bright red colour (life).	Deraniyagala (1930); Pethiyagoda (1991); Talwar and Jhingran (1991).		EUN230<br>RC0053<br>RC0054<br>RC0103<br>RC0141<br>RC0709
Puntius vittatus Day	Barbels absent; mouth terminal; last unbranched dorsal ray not serrated; lateral line incomplete (3–4 pored scales); scales with dark base and dotted margins; vertical blotch on dorsal; dark spot at base of caudal; pigmented anus.	Day (1865). citeTalwar1991.	Day (1865) describes and illustrates a fish with "four black spots" on the body viz. "one just before the dorsal, one under its posterior margin, another at the base of the caudal, and the fourth at the base of the anal. The dorsal has a black streak down it . . ." This fish only has three spots (only two on body), so identification may need to be revisited when modern literature is available.	RC0356<br>RC0357<br>RC0358<br>RC0359<br>RC0360<br>RC0650

Rasbora cf. *aurotaenia* Tirant	Barbels absent; symphyseal knob present; 7½ branched dorsal rays; 5½ branched anal rays; last unbranched dorsal ray not serrated; lateral line complete (27+3 pored scales); 4½ scales between lateral line and dorsal origin; 2½ scale rows between lateral line and pelvic origin; dorsal origin closer to eye than caudal base; weak midlateral stripe (1 scale width) from operculum to caudal peduncle, superimposed onto axial streak.	Kottelat (1998, 2001, 2005); Kottelat et al. (1993).	Specimens in poor condition, so identification tentative.	RC0192<br>RC0193
Rasbora bankanensis (Bleeker)	Barbels absent; symphyseal knob present; 7½ branched dorsal rays; 5½ branched anal rays; last unbranched dorsal ray not serrated; lateral line complete (20–22 pored scales); diffuse midlateral stripe superimposed over prominent axial streak; supra-anal stripe; fins unpigmented except prominent anterior subdistal spot on anal.	Siebert (1997).	Much variation in the size and position of the anal spot between batches. Perhaps a complex of species?	EUN012<br>EUN053<br>EUN203<br>RC0283<br>RC0284<br>YGN124
Rasbora borapetensis Smith	Barbels absent; symphyseal knob present; 7½ branched dorsal rays; 5½ branched anal rays; last unbranched dorsal ray not serrated; lateral line incomplete (13–14 pored scales); midlateral stripe from operculum to caudal base, yellow iridescent stripe above (life); supra-anal stripe and subpeduncular streak present; caudal base red/orange (life); fins otherwise without colour.	Kottelat (2001); Smith (1934).		RC0591<br>RC0592
Rasbora brigittae Vogt	As *R. merah*, but: midlateral blotch and midlateral stripe confluent; red spots on caudal lobes (life).	Conway (2005); Conway and Kottelat (2011); Kottelat (1991); Kottelat and Vidthayanon (1993).	Characters do not appear consistent between *R. brigittae* and *R. merah*. Some examples of *R. merah* have confluent lateral stripe, but red spots on caudal, and examples of *R. brigittae* have red spots on caudal, but midlateral blotch resembling *R. merah*. Generic assignment follows Tang et al. (2010).	EUN223<br>RC0230<br>RC0231<br>YGN169<br>YGN179
Rasbora brittani (Axelrod)	Barbels absent; symphyseal knob absent; pointed snout; elongate body shape; 15 predorsal scales; 7½ branched dorsal rays; 5½ branched anal rays; last unbranched dorsal ray not serrated; dorsal origin posterior to pelvic; lateral line incomplete (10 pored scales), descending in steps; black spot on caudal base occupying 50% of peduncle.	Axelrod (1976); Kottelat (1991, 2008b); Liao et al. (2010); Tan and Kottelat (2009).	Generic assignment follows Tang et al. (2010).	EUN017<br>RC0636
Rasbora caudimaculata Volz	Barbels absent; symphyseal knob present; 7½ branched dorsal rays; 5½ branched anal rays; last unbranched dorsal ray not serrated; lateral line complete; midlateral stripe present, but v. weak axial streak present; supra-anal stripe confluent with sub-peduncular streak; scale pigments giving distinct reticulated pattern throughout body; caudal with black tips; other fins without markings.	Brittan (1972); Kottelat et al. (1993).		EUN050<br>RC0595<br>RC0596
Rasbora cf. *cheeya* (1) (Liao & Tan)	Barbels absent; body bulky; symphyseal knob present; 7½ branched dorsal rays; 5½ branched anal rays; last unbranched dorsal ray not serrated; lateral line complete, and not arranged in "step-like" pattern; dorsal origin anterior to pelvic origin; 9 predorsal scales; large eye; dark blotch in centre of dorsal, more like a bar; dorsal anterior to blotch, green-yellow colour (life).	Brittan (1972); Duncker (1904); Grant (2002); Liao et al. (2010); Liao and Tan (2011).	A larger fish than *Rasbora dorsiocellata*. Appears similar to *R. macrophthalma* Meinken, a species which should have an abbreviated lateral line. The positions of the dorsal fin as described by Grant (2002) is inconsistent with photographs in that article, so these are not regarded as *R. macrophthalma* until the original description or type material become available. Closest to *Brevibora cheeya*, but differs in predorsal scale count (should be 10–11), shape of dorsal blotch (should be round), and lateral line shape (should be "step-like". Generic assignment follows Tang et al. (2010).	RC0686
Rasbora cf. *cheeya* (2) (Liao & Tan)	Barbels absent; body bulky; symphyseal knob present; 7½ branched dorsal rays; 5½ branched anal rays; last unbranched dorsal ray not serrated; lateral line complete, and not arranged in "step-like" pattern; dorsal origin anterior to pelvic origin; 9 predorsal scales; large eye; dark blotch in dorsal; fine, dark granulated chromatophores scattered evenly on head, body and fins.	Brittan (1972); Duncker (1904); Grant (2002); Liao et al. (2010); Liao and Tan (2011).	Specimens in poor condition, but closest to *Brevibora cheeya*. Differs, however, in predorsal scale count (should be 10–11) and lateral line shape (should be "step-like"). Not regarded as conspecific to RC0686 due to distinct pigment colour pattern on body and fins. Generic assignment follows Tang et al. (2010).	YGN431<br>EUN204
Rasbora cf. *dandia* (Valenciennes)	Barbels absent; symphyseal knob not pronounced; mouth terminal; 7½ branched dorsal rays; 5½ branched anal rays; last unbranched dorsal ray not serrated; lateral line complete (28–30 pored scales); ½, 4, 1, 1½ scales in transverse line between dorsal and pelvic origin; 13 predorsal scales; midlateral dark stripe greater than one scale width on caudal peduncle, and extending to median caudal rays; greenish lateral stripe above dark stripe (life).	Kottelat (1998, 2001); Silva et al. (2010).	Identification tentative. Does not conform to *R. daniconius* (Hamilton) s.s., but could be conspecific with Indochinese *R. daniconius* s.l. However, does not disagree with diagnosis of *D. dandia*, and so the name is used here conservatively in the absence of information on Indochinese *R. daniconius*.	RC0651<br>RC0652

Rasbora dorsiocellata Duncker	Barbels absent; body slender; symphyseal knob present; 7½ branched dorsal rays; 5½ branched anal rays; 10–11 predorsal scales; dorsal origin approx. above pelvics; last unbranched dorsal ray not serrated; lateral line incomplete (7–8 pored scales), arranged in "step-like" pattern (see comments); round, dark blotch in centre of dorsal, not reaching last 2 branched rays, not bar-like.	Brittan (1972); Duncker (1904); Grant (2002); Liao et al. (2010); Liao and Tan (2011).	Liao et al. (2010) reports symphyseal knob absent. The "step-like" pattern of the pored lateral line scales was not clear in all specimens (some damaged), with variation apparent. Generic assignment follows Tang et al. (2010).	EUN051<br>RC0291<br>RC0663
Rasbora dusonensis (Bleeker)	Barbels absent; mouth subterminal; symphyseal knob present; 7½ branched dorsal rays; 5½ branched anal rays; last unbranched dorsal ray not serrated; lateral line complete (26+3 pored scales); 10–11 predorsal scales; dorsal origin posterior to pelvic origin; 4½ scales between lateral line and dorsal origin; 1½ scale rows between lateral line and pelvic origin; 3 scale rows between lateral line and mid-ventral row; dorsal origin closer to eye than caudal base; diffuse midlateral stripe from operculum to caudal peduncle; axial streak ventral to midlateral stripe; weak black posterior margin to caudal.	Kottelat (1998, 2001, 2005); Kottelat et al. (1993).		RC0419
Rasbora einthovenii (Bleeker)	Barbels absent; symphyseal knob present on lower jaw; 7½ branched dorsal rays; 5½ branched anal rays; last unbranched dorsal ray not serrated; lateral line complete (28+2 pored scales); uneven, ventrally curved lateral stripe from snout to end of median caudal rays; reticulated scale pattern on dorso-anterior of body; purple hue (life).	Brittan (1972); Kottelat et al. (1993); Tan (2009).		RC0363<br>RC0364
Rasbora cf. *ennealepis* Roberts	Barbels absent; symphyseal knob present; 7½ branched dorsal rays; 5½ branched anal rays; last unbranched dorsal ray not serrated; lateral line complete (26–27 pored scales); 10–11 predorsal scales; 2 rows of scales between lateral line and pelvic origin; caudal peduncle narrow; wide midlateral stripe (2 scales width), more intense posteriorly and superimposed over axial streak; precaudal spot absent; supra-anal stripe present; reticulate pattern weak; anterior anal rays weakly pigmented.	Kottelat (2000); Kottelat et al. (1993); Roberts (1989); Siebert (1997); Siebert and Guiry (1996).	Poor match to *R. ennealepis*, a species with 24–25 pored lateral line scales, 9 predorsal scales and a strongly reticulated scale pattern (Roberts, 1989). Roberts (1989) reported a sample from the Kapuas drainage with 10–11 predorsal scales and a lighter pattern. He regarded these as *R.* cf. *ennealepis*.	RC0660<br>RC0661
Rasbora espei Meinken	As *R. heteromorpha*, but: slimmer, less deep bodied; triangular, posterior black stripe smaller, markedly concave ventrally, forming distinct "lambchop" shape.	Brittan (1972); Duncker (1904); Kottelat et al. (1993); Kottelat and Witte (1999); Meinken (1956).	Generic assignment follows Tang et al. (2010).	EUN054<br>EUN235<br>RC0202<br>RC0496<br>RC0508<br>RC0509<br>YGN280<br>YGN282<br>YGN448
Rasbora gracilis Kottelat	Barbels absent; symphyseal knob absent; slender body shape; pointed snout; triangular-shaped operculum; 7½ branched dorsal rays; 5½ branched anal rays; last unbranched dorsal ray not serrated; dorsal high and strongly pointed; lateral line incomplete (0–4 pored scales); anal concave with elongated anterior rays; conspicuous, wide midlateral stripe continuing onto caudal; slender caudal peduncle.	Kottelat (1991); Liao et al. (2010).	Generic assignment follows Tang et al. (2010).	YGN117<br>YGN432
Rasbora hengeli Meinken	As *R. heteromorpha*, but: slimmer, less deep bodied; triangular, posterior black stripe markedly smaller: distance between pelvic origin and lower anterior edge of stripe equal to greatest width of stripe; colouration generally muted, with grey background colour and bright orange stripe above lateral stripe (life).	Brittan (1972); Duncker (1904); Kottelat et al. (1993); Kottelat and Witte (1999); Meinken (1956).	Generic assignment follows Tang et al. (2010).	YGN480
Rasbora heteromorpha Duncker	Barbels absent; symphyseal knob present on lower jaw; 7½ branched dorsal rays; 5½ branched anal rays; last unbranched dorsal ray not serrated; deep body, strongly laterally compressed; convex body (back) shape posterior to occiput; lateral line incomplete (up to 8 pored scales); conspicuous black stripe commencing posterior to dorsal origin, broader anteriorly covering most of body as triangle, or wedge shape, not concave ventrally; dark pigmentation to anterior dorsal and anal rays; pink/orange/red background colour to body (life).	Brittan (1972); Duncker (1904); Kottelat et al. (1993); Kottelat and Witte (1999); Meinken (1956).	Generic assignment follows Tang et al. (2010).	EUN236<br>RC0308<br>RC0597<br>YGN460<br>YGN506
Rasbora cf. *heteromorpha* Duncker	As *R. heteromorpha*, but: more slender, lacking convexity posterior to occiput; pigmentation on anterior dorsal/anal rays less distinct; orange/yellow anterior-subdistal blotch in anal.	Brittan (1972); Duncker (1904); Kottelat et al. (1993); Kottelat and Witte (1999); Meinken (1956).	Possibly an undescribed species. Generic assignment follows Tang et al. (2010).	RC0201<br>RC0307<br>YGN496
Rasbora kalochroma (Bleeker)	Barbels absent; symphyseal knob present on lower jaw; 7½ branched dorsal rays; 5½ branched anal rays; last unbranched dorsal ray not serrated; red colouration (life); two midlateral blotches (above pectoral and anal); no blotch on peduncle; indistinct posterior stripe from second blotch to end of median caudal rays.	Lim (1995); Tan (2009).		RC0450<br>RC0451<br>YGN133<br>YGN170<br>YGN377

Rasbora maculata Duncker	Barbels absent; scales present; lateral line absent; symphyseal knob weak or absent; miniature size; slender caudal peduncle; 7½ branched dorsal rays; 5½ branched anal rays; 10+9 principal caudal rays; dark lateral blotch anterior to pelvics (larger than pupil); black spot at caudal base; red and black pigmentation on anterior of dorsal and anal (life); conspicuous pigmentation absent between eye and maxilla.	Conway (2005); Conway and Kottelat (2011); Kottelat (1991); Kottelat and Vidthayanon (1993).	Generic assignment follows Tang et al. (2010).	RC0228<br>RC0229<br>YGN132<br>YGN178
Rasbora merah Kottelat	Barbels absent; scales present; lateral line absent; symphyseal knob weak or absent; miniature size; slender caudal peduncle; 7½ branched dorsal rays; 5½ branched anal rays; 7 pelvic rays; oval, longitudinally elongate midlateral blotch between pectoral and pelvic origin (surrounded by area free of pigment); irregular midlateral stripe from above anal origin to caudal peduncle; supra-anal spot; black spot on caudal base; black spot at caudal base; red spot on anterior of dorsal (life); conspicuous pigmentation absent between eye and maxilla; last unbranched anal ray pigmented; red colouration to body (life).	Conway (2005); Conway and Kottelat (2011); Kottelat (1991); Kottelat and Vidthayanon (1993).	See comments for *R. brigittae*. Generic assignment follows Tang et al. (2010).	RC0226<br>RC0227<br>YGN123
Rasbora naevus	As *R. maculata*, but: 9+8 principal caudal rays; sexually dimorphic lateral blotch (smaller in females).	Conway (2005); Conway and Kottelat (2011); Kottelat (1991); Kottelat and Vidthayanon (1993).	Generic assignment follows Tang et al. (2010). Conway and Kottelat (2011) report specimens of *Boraras* cf. *micros* in Tang et al. (2010) (GenBank EF452885 & HM224235) correspond to *R. naevus*.	RC0224<br>RC0225
Rasbora pauciperforata Weber & de Beaufort	Barbels absent; symphyseal knob not distinct; slender body shape; pointed snout; triangular-shaped operculum; 7½ branched dorsal rays; 5½ branched anal rays; last unbranched dorsal ray not serrated; lateral line incomplete (6 pored scales); anal concave with elongated anterior rays; midlateral stripe ending at caudal base, with lighter red stripe above (life); series vertical streaks on anterior scales below midlateral stripe; supra-anal stripe and subpeduncular streak confluent.	Brittan (1972); Kottelat (1991); Kottelat et al. (1993); Liao et al. (2010); Weber and de Beaufort (1916).	Liao et al. (2010) reports symphyseal supra-anal stripe and subpeduncular streak absent. Generic assignment follows Tang et al. (2010).	RC0240<br>RC0241<br>YGN116<br>YGN290
Rasbora cf. *paucisqualis* Ahl	Barbels absent; symphyseal knob present; 7½ branched dorsal rays; 5½ branched anal rays; last unbranched dorsal ray not serrated; lateral line incomplete (13–14 pored scales); no dorsal tubercles; elongate body; midlateral stripe diffuse anteriorly, ventral to axial streak anteriorly, becoming intense posteriorly and ending on caudal base; width of midlateral stripe 1½ scale rows; no precaudal spot; supra-anal stripe distinct; reticulate pattern weak, fins with no colouration.	Kottelat (2000, 2001, 2008b); Siebert (1997); Siebert and Guiry (1996).	*Rasbora paucisqualis* should have 22–27 pored lateral line scales (Siebert, 1997), so have conservatively named these fish *R.* cf. *paucisqualis*.	EUN032<br>EUN229<br>RC0255<br>RC0256
Rasbora paviana Tirant	Barbels absent; symphyseal knob present; 7½ branched dorsal rays; 5½ branched anal rays; last unbranched dorsal ray not serrated; lateral line complete; distinct midlateral stripe starting at operculum, narrow anteriorly (½ scale row width), terminating in contiguous diamond-shaped blotch on caudal base; axial streak superimposed on midlateral stripe for much of length; weak supra-anal pigments; fins without markings.	Kottelat (1998, 2001, 2005).		RC0194<br>RC0195
Rasbora rasbora (Hamilton)	Barbels absent; symphyseal knob present; mouth terminal; 7½ branched dorsal rays; 5½ branched anal rays; last unbranched dorsal ray not serrated; lateral line complete; weak supra-anal stripe; diffuse lateral stripe from operculum to caudal base; subpeduncular streak present; scale pigments giving weak reticulated pattern; caudal yellow (life) with black lobes and posterior margin.	Brittan (1972); Hamilton (1822); Silva et al. (2010).		RC0191<br>RC0513<br>RC0514
Rasbora rubrodorsalis Donoso-Büchner & Schmidt	As *R. borapetensis*, but with: (7–8 pored lateral line scales); red/orange blotch on anterior dorsal base (life).	Kottelat (2001).		RC0630<br>RC0631
Rasbora sarawakensis Brittan	Barbels absent; symphyseal knob present; 7½ branched dorsal rays; 5½ branched anal rays; last unbranched dorsal ray not serrated; body depth 30% in SL; lateral line complete (25 pored scales); tubercles present on dorsal surface; midlateral stripe distinct and of even intensity throughout; supra-anal stripe distinct; subpeduncular streak absent; dorsal and anal fins with dark pigmentation to anterior rays.	Brittan (1972); Kottelat et al. (1993); Roberts (1989).		RC0632<br>RC0633
Rasbora sp. "undet. (1)"	Barbels absent; symphyseal knob present; 7½ branched dorsal rays; 5½ branched anal rays; last unbranched dorsal ray not serrated; lateral line complete; midlateral stripe from operculum to caudal peduncle, widest under dorsal, and terminating in triangular spot; axial streak above, but not separate from midlateral stripe until anterior to anal origin; supra-anal stripe present; distinct reticulate scale pattern; caudal yellow (life) with black tips and thin posterior margin.	Kottelat (1998, 2001, 2005); Kottelat et al. (1993); Tan and Kottelat (2009).	Likely member of the *R. sumatrana* group. Similar to *R. vulgaris* Duncker, *R. notura* Kottelat and *R. hosii* Boulenger, but cannot confidently match due to differences in midlateral stripe arrangement.	RC0574<br>RC0575
Rasbora trilineata Steindachner	Barbels absent; symphyseal knob present; 7½ branched dorsal rays; 5½ branched anal rays; last unbranched dorsal ray not serrated; lateral line complete; midlateral stripe fading anteriorly and widening posteriorly; supra-anal stripe confluent with sub-peduncular streak; scale pigments giving weak reticulated pattern (anteriorly); caudal with oblique subterminal bars and white tips.	Brittan (1972); Kottelat et al. (1993); Rainboth and Kottelat (1987); Roberts (1989).		RC0205<br>RC0206
Rasbora urophthalmoides Kottelat	Barbels absent; scales present; lateral line absent; symphyseal knob weak or absent; minature size (up to 12.4 mm SL); slender caudal peduncle; 7½ branched dorsal rays; 5½ branched anal rays; midlateral stripe from operculum to caudal peduncle; black spot at caudal base; conspicuous pigmentation present between eye and maxilla; last unbranched dorsal ray pigmented; red spots on caudal lobes absent (life).	Conway (2005); Conway and Kottelat (2011); Kottelat (1991); Kottelat and Vidthayanon (1993).	Generic assignment follows Tang et al. (2010).	RC0232<br>RC0233

Rasbora vulcanus Tan	Barbels absent; symphyseal knob present; 7½ branched dorsal rays; 5½ branched anal rays; last unbranched dorsal ray not serrated; lateral line complete; 10 predorsal scales; midlateral stripe from operculum to caudal base; supra-anal stripe and subpeduncular streak present; dorsal, anal and caudal with weak subdistal dark margins; axial streak not distinct; distinct reticulate scale pattern; golden orange colour of body and fins (life).	Tan (1999).		RC0279<br>RC0588<br>YGN034<br>YGN182<br>YGN342
Rasbora wilpita Kottelat & Pethiyagoda	Barbels absent; symphyseal knob pronounced; well developed lateral maxillary process; body depth 25–28% in SL; 7½ branched dorsal rays; 5½ branched anal rays; last unbranched dorsal ray not serrated; lateral line complete (29–31 pored scales); ½, 4, 1, 1½ scales in transverse line between dorsal and pelvic origin; 13 predorsal scales; midlateral dark stripe greater than one scale width on caudal peduncle; upper margin of stripe distinct, with lower margin indistinct giving jagged appearance.	Silva et al. (2010).		RC0285<br>RC0584
Rasboroides vaterifloris (Deraniyagala)	Barbels absent; symphyseal knob present; deep laterally compressed body shape; 7½ branched dorsal rays; 6½ branched anal rays; last unbranched dorsal ray not serrated; lateral line incomplete (up to 3 pored scales); anal strongly concave with rays elongated anteriorly; orange colour of body and fins, with caudal hyaline and orange lower lobe (life).	Brittan (1972); Deraniyagala (1930); Pethiyagoda (1991).		EUN048<br>RC0281<br>RC0282
Rhodeus ocellatus (Kner)	Barbels absent; anal origin before end of dorsal base; lateral line incomplete (up to 4 pored scales); 12½ branched dorsal and anal rays; posterior midlateral stripe, starting after pelvic base; caudal with red median stripe (life); white anterior margin of pelvics (life); 2 rows of white spots along median dorsal rays (life).	Arai and Akai (1988); Nakabo (2002).	Conforms to *R. ocellatus ocellatus*.	RC0572<br>RC0573
Rohtee ogilbii Sykes	Barbels absent; lateral line complete; 8½ branched dorsal rays; 13½ branched anal rays; last unbranched dorsal ray serrated; ventral edge of body sharp and keel-like between pelvics and anal; procumbent predorsal spine (concealed by scales); body deep and laterally compressed; silvery colour (life) with 5 black bars; spot on caudal peduncle.	Day (1865); Sykes (1839, 1841); Talwar and Jhingran (1991).	Matches Talwar and Jhingran (1991) and Day (1865) well, but Sykes (1839) does not mention black bars. Specimen may be a juvenile.	RC0609
Sawbwa resplendens Annandale	Barbels absent; scales absent; last unbranched dorsal ray serrated; 7½ branched dorsal rays; 5½ branched anal rays; body with scattered chromatophores.	Annandale (1918).		EUN173<br>RC0161<br>RC0162<br>YGN396
Sundadanio cf. *axelrodi* (Brittan)	Barbels absent; lateral line absent; symphyseal knob present; head blunt; caudal peduncle slender; miniature size (up to 20 mm TL); 6½ branched dorsal rays; 5½ branched anal rays; posterior margin of anal concave; sexually dichromatic, males with more intense colouration.	Brittan (1976); Kottelat and Witte (1999); Roberts (1989).	Sold in aquarium trade as three colour varieties: red, blue, green. Likely a complex of species. Mostly female specimens here, so hard to characterise diagnostic male colour patterns and match specimens to type material, so all regarded here as *S.* cf. *axelrodi*.	EUN099<br>EUN231<br>RC0236<br>RC0237<br>RC0238<br>RC0239<br>YGN073<br>YGN119<br>YGN120<br>YGN121
Tanakia himantegus (Günther)	One pair barbels (greater than eye diameter); anal origin before end of dorsal base; lateral line complete; 8½ branched dorsal rays; 10½ branched anal rays; median row of elongated spots on dorsal membrane; anal with black distal stripe and red median stripe (life); midlateral stripe starting above pelvis base, widening posteriorly and continuing onto caudal; red distal band on dorsal (life); upper of iris red (life); midlateral spot above pectoral.	Arai and Akai (1988); Chang et al. (2009); Günther (1868); Nakabo (2002).	Conforms to *T. himantegus himantegus*.	RC0466<br>RC0467
Tanichthys albonubes Lin	Barbels absent; symphyseal knob absent; lateral line absent; posterior and anterior nostrils confluent; 6½ branched dorsal rays; 8½ branched anal rays; row cornified tubercles on snout of male; dark midlateral stripe terminating as spot on caudal base, with light stripe above; dark stripe narrower than light stripe; distance between dorsal origin and top of light stripe half of distance between anal origin and bottom of dark stripe; body below dark midlateral stripe dark coloured; dusky caudal with red blotch at centre and base (life).	Freyhof and Herder (2001); Liang et al. (2008); Weitzman and Chan (1966).		EUN234<br>RC0442<br>RC0449
Tanichthys micagemmae Freyhof & Herder	As *T. albonubes*, but: dark midlateral stripe wider than light midlateral stripe; distance between dorsal origin roughly equal or greater than distance between anal origin and dark stripe; body below dark midlateral stripe light coloured.	Freyhof and Herder (2001); Liang et al. (2008); Weitzman and Chan (1966).	Tubercles not observed in these specimens, as all female.	EUN011<br>RC0478<br>RC0479<br>YGN259<br>YGN420

References

Ahl, E. (1929). Übersicht über die lebend eingeführten asiatischen arten der gattung *Barbus*. *Das Aquarium*, 1929(Oct.):165–169.

Alfred, E. R. (1963). Some colourful fishes of the genus *Puntius* Hamilton. *Bulletin of the Singapore National Museum*, 32:135–142.

Annandale, N. (1918). Fish and fisheries of the Inlé Lake. *Records of the Indian Museum*, 14:33–64.

Arai, R. and Akai, Y. (1988). *Acheilognathus melanogaster*, a senior synonym of *A. moriokae*, with a revision of the genera of the subfamily Acheilognathinae (Cypriniformes, Cyprinidae). *Bulletin of the National Science Museum, Tokyo, Series A*, 14:199–213.

Arunkumar, L. and Tombi Singh, H. (2003). Two new species of puntiid fish from the Yu River system of Manipur. *Journal of the Bombay Natural History Society*, 99(3):481–487.

Axelrod, H. R. (1976). *Rasbora brittani*, a new species of cyprinid fish from the Malay Peninsula. *Tropical Fish Hobbyist*, 24(6):94–98.

Banarescu, P. (1986). A review of the species of *Crossocheilus, Epalzeorhynchos* and *Paracrossochilus* (Pisces, Cyprinidae). *Travaux du Museum d'Histoire Naturelle*, 28:141–161.

Barman, R. P. (1984a). A new freshwater fish of the genus *Danio* Hamilton (Pisces: Cyprinidae) from Assam, India, with the key to the identification of the Indian species of the subgenus *Danio*. *Bulletin of the Zoological Survey of India*, 6:163–165.

Barman, R. P. (1984b). On a new species of the genus *Danio* Hamilton from Burma (Pisces: Cyprinidae). *Bulletin of the Zoological Survey of India*, 5(2-3):31–34.

Barman, R. P. (1991). A taxonomic revision of the Indo-Burmese species of *Danio* Hamilton Buchanan (Pisces: Cyprinidae). *Records of the Zoological Survey of India*, 137:1–91.

Bordoloi, S. and Baishya, A. (2006). *Puntius ornatus* from the Brahmaputra drainage in Assam. *Zoos' Print Journal*, 21(6):2292–2294.

Boschung, H. T. and Mayden, R. L. (2004). *Fishes of Alabama*. Smithsonian Institution Press, Washington.

Boulenger, G. A. (1907). Descriptions of three new freshwater fishes discovered by Mr. G. L. Bates in South Cameroon. *Annals and Magazine of Natural History*, 20(120):485–487.

Brittan, M. R. (1972). *A revision of the Indo-Malayan fresh-water fish genus* Rasbora. T.F.H. Publications, Neptune.

Brittan, M. R. (1976). *Rasbora axelrodi*, a new cyprinid from Indonesia. *Tropical Fish Hobbyist*, 25(4):92–98.

Britz, R. (2009). *Danionella priapus*, a new species of miniature cyprinid fish from West Bengal, India (Teleostei: Cypriniformes: Cyprinidae). *Zootaxa*, 2277:53–60.

Britz, R., Conway, K. W., and Rüber, L. (2009). Spectacular morphological novelty in a miniature cyprinid fish, *Danionella dracula* n. sp. *Proceedings of the Royal Society B*, 276(1665):2179–2186.

Britz, R. and Kottelat, M. (2008). *Paedocypris carbunculus*, a new species of miniature fish from Borneo (Teleostei: Cypriniformes: Cyprinidae). *Raffles Bulletin of Zoology*, 56(2):415–422.

Chang, C., Lin, W. W., Shao, Y. T., Arai, R., Ishinabe, T., Ueda, T., Matsuda, M., Kubota, H., Wang, F. Y., and Jang-Liaw, N. H. (2009). Molecular phylogeny and genetic differentiation of the *Tanakia himantegus* complex (Teleostei: Cyprinidae) in Taiwan and China. *Zoological Studies*, 48(6):823–834.

Chang, C., Shao, Y. T., and Kao, H. W. (2006). Molecular identification of two sibling species of *Puntius* in Taiwan. *Zoological Studies*, 45(2):149–156.

Chen, X.-Y., Yang, J.-X., and Chen, Y.-R. (1999). A review of the cyprinoid fish genus *Barbodes* Bleeker, 1859, from Yunnan, China, with descriptions of two new species. *Zoological Studies*, 38(1):82–88.

Coad, B. (2010). *Freshwater Fishes of Iran*. World Wide Web electronic publication: `http://www.briancoad.com/Species Accounts/Cyprinidae Garra to Vimba.htm`.

Conway, K. W. (2005). Monophyly of the genus *Boraras* (Teleostei: Cyprinidae). *Ichthyological Exploration of Freshwaters*, 16(3):249–264.

Conway, K. W., Chen, W. J., and Mayden, R. L. (2008). The "celestial pearl danio" is a miniature *Danio* (s.s.) (Ostariophysi: Cyprinidae): evidence from morphology and molecules. *Zootaxa*, 1686:1–28.

Conway, K. W. and Kottelat, M. (2011). *Boraras naevus*, a new species of miniature and sexually dichromatic freshwater fish from peninsular Thailand (Ostariophysi: Cyprinidae). *Zootaxa*, 3002:45–51.

Conway, K. W., Mayden, R. L., and Tang, K. L. (2009). *Devario anomalus*, a new species of freshwater fish from Bangladesh (Ostariophysi: Cyprinidae). *Zootaxa*, 58:49–58.

Conway, K. W. and Moritz, T. (2006). *Barboides britzi*, a new species of minature cyprinid from Benin (Ostariophysi: Cyprinidae), with a neotype designation for *B. gracilis*. *Ichthyological Exploration of Freshwaters*, 17(1):73–84.

Cottle, P. W. (2010). *Danios and Devarios*. Peter W. Cottle, Rochester.

Day, F. (1865). *The fishes of Malabar*. Bernard Quaritch, London.

Day, F. (1870). Notes on some fishes from the western coast of India. *Proceedings of the General Meetings for Scientific Business of the Zoological Society of London*, 1870(2):369–374.

Day, F. (1875). *The fishes of India; being a natural history of the fishes known to inhabit the seas and fresh waters of India, Burma, and Ceylon*. Bernard Quaritch, London.

Deraniyagala, P. E. P. (1930). The Eventognathi of Ceylon. *The Ceylon Journal of Science*, 16(1):1–41.

Devi, K. R., Indra, T. J., and Knight, J. D. M. (2010). *Puntius rohani* (Teleostei: Cyprinidae), a new species of barb in the *Puntius filamentosus* group from the southern Western Ghats of India. *Journal of Threatened Taxa*, 2(9):1121–1129.

Doi, A. and Taki, Y. (1994). A new cyprinid fish, *Hampala salweenensis*, from the Mae Pai river system, Salween basin, Thailand. *Japanese Journal of Ichthyology*, 40(4):405–412.

Duncker, G. (1904). Die Fische der malayischen Halbinsel. *Mitteilungen aus dem Naturhistorischen (Zoologischen)*, 21:133–207.

Fang, F. (1997a). *Danio maetaengensis*, a new species of cyprinid fish from northern Thailand. *Ichthyological Exploration of Freshwaters*, 8(1):41–48.

Fang, F. (1997b). Redescription of *Danio kakhienensis*, a poorly known cyprinid fish from the Irrawaddy basin. *Ichthyological Exploration of Freshwaters*, 7(4):289–298.

Fang, F. (1998). *Danio kyathit*, a new species of cyprinid fish from Myitkyina, northern Myanmar. *Ichthyological Exploration of Freshwaters*, 8(3):273–280.

Fang, F. (2000). A review of Chinese *Danio* species (Teleostei: Cyprinidae). *Acta Zootaxonomica Sinica*, 25(2):214–227.

Fang, F. (2003). Phylogenetic analysis of the Asian cyprinid genus *Danio* (Telostei, Cyprinidae). *Copeia*, 2003(4):714–728.

Fang, F. and Kottelat, M. (1999). *Danio* species from northern Laos, with descriptions of three new species (Teleostei: Cyprinidae). *Ichthyological Exploration of Freshwaters*, 10(3):281–295.

Fang, F. and Kottelat, M. (2000). *Danio roseus*, a new species from the Mekong basin in northeastern Thailand and northwestern Laos (Teleostei: Cyprinidae). *Ichthyological Exploration of Freshwaters*, 11(2):149–154.

Fang, F. and Kullander, S. O. (2009). *Devario xyrops*, a new species of danionine fish from south-western Myanmar (Teleostei: Cyprinidae). *Zootaxa*, 2164:33–40.

Fang, F., Norén, M., Liao, T. Y., Källersjö, M., and Kullander, S. O. (2009). Molecular phylogenetic interrelationships of the south Asian cyprinid genera *Danio, Devario* and *Microrasbora* (Teleostei, Cyprinidae, Danioninae). *Zoologica Scripta*, 38(3):237–256.

Fowler, H. W. (1934). Zoological results of the third De Schauensee Siamese Expedition, Part V.–Additional fishes. *Proceedings of the Academy of Natural Sciences of Philadelphia*, 86:335–352.

Fowler, H. W. (1935). Zoological results of the third De Schauensee Siamese Expedition, Part VI.–Fishes obtained in 1934. *Proceedings of the Academy of Natural Sciences of Philadelphia*, 87:89–163.

Freyhof, J. and Herder, F. (2001). *Tanichthys micagemmae*, a new miniature cyprinid fish from Central Vietnam (Cypriniformes: Cyprinidae). *Ichthyological Exploration of Freshwaters*, 12(3):215–220.

Gante, H. F., Moreira Da Costa, L., Micael, J., and Alves, M. J. (2008). First record of *Barbonymus schwanenfeldii* (Bleeker) in the Iberian Peninsula. *Journal of Fish Biology*, 72(4):1089–1094.

Gao, Z., Li, Y., and Wang, W. (2008). Threatened fishes of the world: *Myxocyprinus asiaticus* Bleeker 1864 (Catostomidae). *Environmental Biology of Fishes*, 83(3):345–346.

Grant, S. (2002). Zur Identität und Gültigkeit von *Rasbora macrophthalma* Meinken, 1951 (Cyprinidae: Rasborinae). *BSSW-Report, Verband Deutscher für Aquarien- und Terrarienkunde*, 2002(4):13–17.

Günther, A. (1868). Catalogue of the fishes in the British Museum. *Catalogue of the fishes in the British Museum*, 7:1–512.

Hamilton, F. (1822). *An account of the fishes found in the river Ganges and its branches.* George Ramsay and Co, Edinburgh.

Herre, A. (1940). Additions to the fish fauna of Malaya and notes on rare or little known Malayan and Bornean fishes. *Bulletin of the Raffles Museum*, 16:27–61.

Hopson, A. J. (1965). *Barbus* (Pisces, Cyprinidae) of the Volta region. *Bulletin of the British Museum (Natural History)*, 13(4):126–128.

Hora, S. L. (1921). Fish and fisheries of Manipur with some observations on those of the Naga Hills. *Records of the Indian Museum*, 22(3):165–214.

Hora, S. L. (1928). Notes on fishes in the Indian Museum. XV.– Notes on Burmese fishes. *Records of the Indian Museum*, 30(1):37–40.

Hora, S. L. (1937). On a small collection of fish from Sandoway, Lower Burma. *Records of the Indian Museum*, 39(4):323–331.

Hora, S. L. and Mukerji, D. D. (1928). Notes on fishes in the Indian Museum. XVI.–On fishes of the genus *Esomus* Swainson. *Records of the Indian Museum*, 30:41–56.

Hora, S. L. and Mukerji, D. D. (1934). On the collection of fish from the S. Shan states and the Pegu Yomas, Burma. *Records of the Indian Museum*, 36(1):123–138.

Inger, R. F. and Chin, P. K. (1962). The fresh-water fishes of North Borneo. *Fieldiana Zoology*, 45:1–268.

Jayaram, K. C. (1990). Two new species of the genus *Puntius* Hamilton (Pisces: Cyprinidae) from India. *Journal of the Bombay Natural History Society*, 87(1):106–109.

Jayaram, K. C. (1991). Systematic status of *Danio malabaricus* (Pisces: Cyprinidae). *Ichthyological Explorations of Freshwaters*, 2(11):109–112.

Jerdon, T. C. (1849). On the fresh-water fishes of southern India. *Madras Journal of Literature and Science*, 15(2):302–346.

Jiang, Y. E., Chen, X. Y., and Yang, J. X. (2008). *Microrasbora* Annandale, a new genus record in China, with description of a new species (Teleostei: Cyprinidae). *Environmental Biology of Fishes*, 83(3):299–304.

Knight, J. D. M., Devi, K. R., and Atkore, V. (2011). Systematic status of *Systomus rubrotinctus* Jerdon (Teleostei: Cyprinidae) with notes on the *Puntius arulius* group of fishes. *Journal of Threatened Taxa*, 3(4):1686–1693.

Kottelat, M. (1982). A small collection of fresh-water fishes from Kalimantan, Borneo, with descriptions of one new genus and three new species of Cyprinidae. *Revue Suisse de Zoologie*, 89(2):419–437.

Kottelat, M. (1991). Notes on the taxonomy of some Sundaic and Indochinese species of *Rasbora*, with description of four new species (Pisces: Cyprinidae). *Ichthyological Exploration of Freshwaters*, 2(2):177–191.

Kottelat, M. (1996). The identity of *Puntius eugrammus* and diagnoses of two new species of striped barbs (Teleostei: Cyprinidae) from Southeast Asia. *The Raffles Bulletin of Zoology*, 44(1):301–316.

Kottelat, M. (1998). Fishes of the Nam Theun and Xe Bangfai basins, Laos, with diagnoses of twenty-two new species (Teleostei: Cyprinidae, Balitoridae, Cobitidae, Coiidae and Odontobutidae). *Ichthyological Exploration of Freshwaters*, 9(1):1–128.

Kottelat, M. (2000). Diagnoses of a new genus and 64 new species of fishes from Laos (Teleostei: Cyprinidae, Balitoridae, Bagridae, Syngnathidae, Chaudhuriidae and Tetraodontidae). *Journal of South Asian Natural History*, 5(1):37–82.

Kottelat, M. (2001). *Fishes of Laos*. WHT Publications (Pte) Ltd, Colombo.

Kottelat, M. (2005). *Rasbora notura*, a new species of cyprinid fish from the Malay Peninsula (Teleostei: Cyprinidae). *Ichthyological Exploration of Freshwaters*, 16(3):265–270.

Kottelat, M. (2008a). *Osteochilus bleekeri*, a new species of fish from Borneo and Sumatra (Teleostei: Cyprinidae). *Ichthyological Exploration of Freshwaters*, 19(3):249–253.

Kottelat, M. (2008b). *Rasbora dies*, a new species of cyprinid fish from eastern Borneo (Teleostei: Cyprinidae). *Ichthyological Exploration of Freshwaters*, 18(4):301–305.

Kottelat, M., Britz, R., Tan, H. H., and Witte, K. E. (2006). *Paedocypris*, a new genus of Southeast Asian cyprinid fish with a remarkable sexual dimorphism, comprises the world's smallest vertebrate. *Proceedings of the Royal Society B*, 273(1589):895–899.

Kottelat, M. and Freyhof, J. (2007). *Handbook of European freshwater fishes*. Publications Kottelat, Cornol, Switzerland.

Kottelat, M. and Lim, K. K. P. (1995). Freshwater fishes of Sarawak and Brunei Darussalam: a preliminary annotated check-list. *The Sarawak Museum Journal*, 48(69):227–256.

Kottelat, M. and Pethiyagoda, R. (1990). *Danio pathirana*, a new species of cyprinid fish endemic to southern Sri Lanka. *Ichthyological Exploration of Freshwaters*, 1(3):247–252.

Kottelat, M. and Pethiyagoda, R. (1991). *Descriptions of three new species of cyprinid fishes from Sri Lanka*, pages 298–313. Wildlife Heritage Trust of Sri Lanka, Colombo.

Kottelat, M. and Tan, H. H. (2009). *Osteochilus flavicauda*, a new species of fish from the Malay Peninsula (Teleostei: Cyprinidae). *Ichthyological Exploration of Freshwaters*, 20(1):1–5.

Kottelat, M. and Vidthayanon, C. (1993). *Boraras micros*, a new genus and species of minute freshwater fish from Thailand (Teleostei: Cyprinidae). *Ichthyological Exploration of Freshwaters*, 4(2):161–176.

Kottelat, M., Whitten, A. J., Kartikasari, S. N., and Wirjoatmodjo, S. (1993). *Freshwater fishes of Western Indonesia and Sulawesi*. Periplus Editions, Hong Kong.

Kottelat, M. and Widjanarti, E. (2005). The fishes of Danau Sentarum National Park and the Kapuas Lakes Area, Kalimantan Barat, Indonesia. *Raffles Bulletin of Zoology Supplement*, 13:139–173.

Kottelat, M. and Witte, K. E. (1999). Two new species of *Microrasbora* from Thailand and Myanmar, with two new generic names for small Southeast Asian cyprinid fishes (Teleostei: Cyprinidae). *Journal of South Asian Natural History*, 4(1):49–56.

Kullander, S. O. (2008). Five new species of *Puntius* from Myanmar (Teleostei: Cyprinidae). *Ichthyological Exploration of Freshwaters*, 19(1):59–84.

Kullander, S. O. and Britz, R. (2008). *Puntius padamya*, a new species of cyprinid fish from Myanmar (Teleostei: Cyprinidae). *Electronic Journal of Ichthyology*, 4(2):56–66.

Kullander, S. O. and Fang, F. (2004). Seven new species of *Garra* (Cyprinidae: Cyprininae) from the Rakhine Yoma, southern Myanmar. *Ichthyological Exploration of Freshwaters*, 15(3):257–278.

Kullander, S. O. and Fang, F. (2005). Two new species of *Puntius* from northern Myanmar (Teleostei: Cyprinidae). *Copeia*, 2005(2):290–302.

Kullander, S. O. and Fang, F. (2009a). *Danio aesculapii*, a new species of danio from south-western Myanmar (Teleostei: Cyprinidae). *Zootaxa*, 2164:41–48.

Kullander, S. O. and Fang, F. (2009b). *Danio tinwini*, a new species of spotted danio from northern Myanmar (Teleostei: Cyprinidae). *Ichthyological Exploration of Freshwaters*, 20(3):223–228.

Kullander, S. O., Liao, T. Y., and Fang, F. (2009). *Danio quagga*, a new species of striped danio from western Myanmar (Teleostei: Cyprinidae). *Ichthyological Exploration of Freshwaters*, 20(3):193–199.

Liang, X. F., Chen, G. Z., Chen, X. L., and Yue, P. Q. (2008). Threatened fishes of the world: *Tanichthys albonubes* Lin 1932 (Cyprinidae). *Environmental Biology of Fishes*, 82(2):177–178.

Liao, T. Y., Kullander, S. O., and Fang, F. (2010). Phylogenetic analysis of the genus *Rasbora* (Teleostei: Cyprinidae). *Zoologica Scripta*, 39(2):155–176.

Liao, T. Y. and Tan, H. H. (2011). *Brevibora cheeya*, a new species of cyprinid fish from Malay Peninsula and Sumatra. *The Raffles Bulletin of Zoology*, 59(1):77–82.

Lim, K. K. P. (1995). *Rasbora kottelati*, a new species of cyprinid fish from north-western Borneo. *The Raffles Bulletin of Zoology*, 43(1):65–74.

Linthoingambi, I. and Vishwanath, W. (2007). Two new fish species of the genus *Puntius* Hamilton (Cyprinidae) from Manipur, India, with notes on *P. ticto* (Hamilton) and *P. stoliczkanus* (Day). *Zootaxa*, 1450:45–56.

Matthews, W. J. (1987). Geographic variation in *Cyprinella lutrensis* (Pisces: Cyprinidae) in the United States, with notes on *Cyprinella lepida*. *Copeia*, 1987(3):616–637.

Mayden, R. L., Tang, K. L., Conway, K. W., Freyhof, J., Chamberlain, S., Haskins, M., Schneider, L., Sudkamp, M., Wood, R. M., and Agnew, M. (2007). Phylogenetic relationships of *Danio* within the order Cypriniformes: a framework for comparative and evolutionary studies of a model species. *Journal of Experimental Zoology Part B: Molecular and Developmental Evolution*, 308(5):642–654.

McClelland, J. (1839). Indian Cyprinidae. *Asiatic Researches*, 19(2):217–471.

Meinken, H. (1956). Mitteilungen der fischbestimmungsstelle des VDA. XXIII. *Rasbora hengeli* spec. nov., eine sehr hübsche neuheit für das liebhaberbecken. *Aquarien und Terrarien-Zeitschrift*, 9(11):281–283.

Menon, A. G. K. (1952). Notes on fishes in the Indian Museum. XLVI. –On a new fish of the genus *Laubuca* from Cochin. *Records of the Indian Museum*, 49(1):1–4.

Menon, A. G. K. (1964). Monograph of the cyprinid fishes of the genus *Garra* Hamilton. *Memoirs of the Indian Museum*, 14(4):173–260.

Menon, A. G. K., Rema Devi, K., and Thobias, M. P. (1999). *Puntius chalakkudiensis*, a new colourful species of *Puntius* (family: Cyprinidae) fish from Kerala, South India. *Records of the Zoological Survey of India*, 97(4):61–63.

Menon, A. G. K., Rema Devi, K., and Vishwanath, W. (2000). A new species of *Puntius* (Cyprinidae: Cyprininae) from Manipur, India. *Journal of the Bombay Natural History Society*, 97(2):263–268.

Myers, G. S. (1924). On a small collection of fishes from Upper Burma. *American Museum Novitates*, 150:1–7.

Nakabo, T. (2002). *Fishes of Japan with pictorial keys to the species, English edition.* Tokai University Press, Tokyo.

Ng, H. H. and Kottelat, M. (2007). *Balantiocheilos ambusticauda*, a new and possibly extinct species of cyprinid fish from Indochina (Cypriniformes: Cyprinidae). *Zootaxa*, 1463:13–20.

Ng, H. H. and Tan, H. H. (1999). The fishes of the Endau drainage, Peninsular Malaysia with descriptions of two new species of catfishes (Teleostei: Akysidae, Bagridae). *Zoological Studies*, 38(3):350–366.

Pethiyagoda, R. (1991). *Freshwater fishes of Sri Lanka.* The Wildlife Heritage Trust of Sri Lanka, Colombo.

Pethiyagoda, R. and Kottelat, M. (2005). A review of the barbs of the *Puntius filamentosus* group (Teleostei: Cyprinidae) of Southern India and Sri Lanka. *Raffles Bulletin of Zoology Supplement*, 12:127–144.

Pethiyagoda, R., Kottelat, M., Silva, A., Maduwage, K., and Meegaskumbura, M. (2008). A review of the genus *Laubuca* in Sri Lanka, with description of three new species (Teleostei: Cyprinidae). *Ichthyological Exploration of Freshwaters*, 19(1):7–26.

Prasad, G., Ali, A., and Raghavan, R. (2008). Threatened fishes of the world: *Puntius denisonii* (Day 1865)(Cyprinidae). *Environmental Biology of Fishes*, 83(2):189–190.

Rachmatika, I. (2004). A new species of cyprinid fish: *Puntius bunau* from the Seturan basin of Indonesian Borneo. *Treubia*, 33(2):181–190.

Rainboth, W. J. (1996). *Fishes of the Cambodian Mekong.* FAO, Rome.

Rainboth, W. J. and Kottelat, M. (1987). *Rasbora spilocerca*, a new cyprinid from the Mekong river. *Copeia*, 1987(2):417–423.

Regan, C. T. (1907). Description of a new cyprinid fish of the genus *Danio* from upper Burma. *Records of the Indian Museum*, 1(4):395.

Remi Devi, K., Indra, T. J., Raghunathan, M. B., and Raagam, P. M. (2005). A note on *Barilius bakeri* (Cyprinidae: Danioninae) from Karnataka with remarks on the status of *Opsarius malabaricus* Jerdon. *Journal of the Bombay Natural History Society*, 102(1):123–125.

Roberts, T. R. (1986). *Danionella translucida*, a new genus and species of cyprinid fish from Burma, one of the smallest living vertebrates. *Environmental Biology of Fishes*, 16(4):231–241.

Roberts, T. R. (1989). The freshwater fishes of Western Borneo (Kalimantan Barat, Indonesia). *Memoirs of the California Academy of Sciences*, 14:1–210.

Roberts, T. R. (1994). Systematic revision of the Southeast Asian cyprinid fish genus *Labiobarbus* (Teleostei: Cyprinidae). *Raffles Bulletin of Zoology*, 41(2):315–329.

Roberts, T. R. (2007). The celestial pearl danio, a new genus and species of colourful minute cyprinid fish from Myanmar (Pisces: Cypriniformes). *The Raffles Bulletin of Zoology*, 55(1):131–140.

Roberts, T. R. and Kottelat, M. (1993). Revision of the southeast Asian freshwater family Gyrinocheilidae. *Ichthyological Exploration of Freshwaters*, 4(4):375–383.

Ryan, J. R. J. and Esa, Y. B. (2006). Phylogenetic analysis of *Hampala* fishes (subfamily Cyprininae) in Malaysia inferred from partial mitochondrial cytochrome *b* DNA sequences. *Zoological Science*, 23(10):893–901.

Schäfer, F. (2009). *Oreichthys crenuchoides*, a new cyprinid from West Bengal, India. *Ichthyological Exploration of Freshwaters*, 20(3):201–211.

Sen, N. and Dey, S. C. (1985). Two new fish species of the genus *Danio* Hamilton (Pisces: Cyprinidae) from Meghalaya, India. *Journal Assam Scientific Society*, 27(2):60–68.

Siebert, D. J. (1997). The identities of *Rasbora paucisqualis* Ahl in Schreitmüller, 1935, and *Rasbora bankanensis* (Bleeker, 1853), with the designation of a lectotype for *R. paucisqualis* (Teleostei: Cyprinidae). *The Raffles Bulletin of Zoology*, 45(1):29–37.

Siebert, D. J. and Guiry, S. (1996). *Rasbora johannae* (Teleostei: Cyprinidae), a new species of the *R. trifasciata*-complex from Kalimantan, Indonesia. *Cybium*, 20(4):395–404.

Silas, E. G. (1953). Notes on fishes from Mahableshwar and Wai (Satara district, Bombay state). *Journal of the Bombay Natural History Society*, 51(3):579–589.

Silva, A., Maduwage, K., and Pethiyagoda, R. (2008). *Puntius kamalika*, a new species of barb from Sri Lanka (Teleostei: Cyprinidae). *Zootaxa*, 64:55–64.

Silva, A., Maduwage, K., and Pethiyagoda, R. (2010). A review of the genus *Rasbora* in Sri Lanka, with description of two new species (Teleostei: Cyprinidae). *Ichthyological Exploration of Freshwaters*, 21(1):27–50.

Skelton, P. H. (2001). *A complete guide to the freshwater fishes of southern Africa.* Struik Publishers, Cape Town.

Smith, H. M. (1931). Descriptions of new genera and species of Siamese fishes. *Proceedings of the United States National Museum*, 79(2873):1–48.

Smith, H. M. (1934). Contributions to the ichthyology of Siam. *Journal of the Siam Society, Natural History Supplement*, 9(3):287–325.

Sykes, W. H. (1839). On the fishes of the Deccan. *Proceedings of the General Meetings for Scientific Business of the Zoological Society of London*, 1838(6):157–165.

Sykes, W. H. (1841). On the fishes of the Dukhun. *Transactions of the Zoological Society of London*, 2:349–378.

Taki, Y. and Katsuyama, A. (1979). Differentiation and zoogeography of two species of the cyprinid genus *Puntioplites*. *Japanese Journal of Ichthyology*, 26(3):253–265.

Talwar, P. K. and Jhingran, A. G. (1991). *Inland fishes of India and adjacent countries.* Oxford & IBH Publishing Co., New Delhi.

Tan, H. H. (1999). *Rasbora vulcanus*, a new species of cyprinid fish from Central Sumatra. *Journal of South Asian Natural History*, 4(2):111–116.

Tan, H. H. (2009). *Rasbora patrickyapi*, a new species of cyprinid fish from Central Kalimantan, Borneo. *The Raffles Bulletin of Zoology*, 57(2):505–509.

Tan, H. H. and Kottelat, M. (2008). Revision of the cyprinid fish genus *Eirmotus*, with description of three new species from Sumatra and Borneo. *Raffles Bulletin of Zoology*, 56(2):423–433.

Tan, H. H. and Kottelat, M. (2009). The fishes of the Batang Hari drainage, Sumatra, with description of six new species. *Ichthyological Exploration of Freshwaters*, 20(1):13–69.

Tang, K. L., Agnew, M. K., Hirt, M. V., Sado, T., Schneider, L. M., Freyhof, J., Sulaiman, Z., Swartz, E., Vidthayanon, C., Miya, M., Saitoh, K., Simons, A. M., Wood, R. M., and Mayden, R. L. (2010). Systematics of the subfamily Danioninae (Teleostei: Cypriniformes: Cyprinidae). *Molecular Phylogenetics and Evolution*, 57(1):189–214.

Tilak, R. and Jain, S. (1990). Description of a new rasborine fish, *Esomus manipurensis* from Manipur, India. *Journal of the Bombay Natural History Society*, 86(3):408–411.

Tshibwabwa, S. M., Stiassny, M. L. J., and Schelly, R. C. (2006). Description of a new species of *Labeo* (Teleostei: Cyprinidae) from the lower Congo river. *Zootaxa*, 1224:33–44.

Tshibwabwa, S. M. and Teugels, G. G. (1995). Contribution to the systematic revision of the African cyprinid fish genus *Labeo*: species from the Lower Zaire river system. *Journal of Natural History*, 29(6):1543–1579.

Tweedie, M. W. F. (1961). Notes on Malayan fresh water fishes. *Bulletin of the Raffles Museum*, 26:178–181.

Vidthayanon, C. and Kottelat, M. (2003). Three new species of fishes from Tham Phra Wang Daeng and Tham Phra Sai Ngam caves in northern Thailand (Teleostei: Cyprinidae and Balitoridae). *Ichthyological Exploration of Freshwaters*, 14(2):159–174.

Vishwanath, W. and Laisram, J. (2004). Two new species of *Puntius* Hamilton-Buchanan (Cypriniformes: Cyprinidae) from Manipur, India, with an account of *Puntius* species from the state. *Journal of the Bombay Natural History Society*, 101(1):130–137.

Vishwanath, W., Lakra, W. S., and Sarkar, U. K. (2007). *Fishes of North East India.* National Bureau of Fish Genetic Resources, Lucknow.

Weber, M. and de Beaufort, L. F. (1916). The fishes of the Indo-Australian Archipelago. III. Ostariophysi: II Cyprinoidea, Apodes, Synbranchi. *The Fishes of the Indo-Australian Archipelago*, 3:1–455.

Weitzman, S. H. and Chan, L. L. (1966). Identification and relationships of *Tanichthys albonubes* and *Aphyocypris pooni*, two cyprinid fishes from South China and Hong Kong. *Copeia*, 1966(2):285–296.

Yazdani, G. M. and Talukdar, S. (1975). A new species of *Puntius* (Cypriniformes: Cyprinidae) from Khasi and Jaintia Hills (Meghalaya), India. *Journal of the Bombay Natural History Society*, 72(1):218–221.

Zhang, E. and Kottelat, M. (2006). *Akrokolioplax*, a new genus of Southeast Asian labeonine fishes (Teleostei: Cyprinidae). *Zootaxa*, 1225:21–30.
